# Counterion-Mediated Assembly of Fluorocarbon–Hydrocarbon Surfactant Mixtures at the Air–Liquid Interface: A Molecular Dynamics Study

**DOI:** 10.3390/molecules30122592

**Published:** 2025-06-14

**Authors:** Xiaolong Quan, Tong Tong, Tao Li, Dawei Han, Baolong Cui, Jing Xiong, Zekai Cui, Hao Guo, Jinqing Jiao, Yuechang Wei

**Affiliations:** State Key Laboratory of Heavy Oil Processing, China University of Petroleum (Beijing), Beijing 102249, China; qxl15993571310@163.com (X.Q.); tongtong001020@163.com (T.T.); litaoreat@163.com (T.L.); 2021310806@student.cup.edu.cn (D.H.); cbl9990501@163.com (B.C.); xiongjing@cup.edu.cn (J.X.); 2023216138@student.cup.edu.cn (Z.C.); gh15207@outlook.com (H.G.)

**Keywords:** molecular dynamics simulation, surfactant aggregation, counterions, short-chain fluorocarbon surfactants, hydrocarbon surfactants

## Abstract

This study employs molecular dynamics simulations to investigate counterion effects (Li^+^, Na^+^, K^+^) on the interfacial aggregation of mixed short-chain fluorocarbon, Perfluorohexanoic acid (PFH_X_A), and Sodium dodecyl sulfate (SDS) surfactants. Motivated by the need for greener surfactant alternatives and a fundamental understanding of molecular interactions governing their behavior, we demonstrate that counterion hydration radius critically modulates system organization. K^+^ ions induce superior monolayer condensation and interfacial performance compared to Li^+^ and Na^+^ counterparts, as evidenced by threefold analysis: (1) RMSD/MSD-confirmed equilibrium attainment ensures data reliability; (2) 1D/2D density profiles and surface tension measurements reveal K^+^-enhanced packing density (lower solvent-accessible surface area versus Na^+^ and Li^+^ systems); (3) Electrostatic potential analysis identifies synergistic complementarity between SDS’s hydrophobic stabilization via dodecyl chain interactions and PFH_X_A’s charge uniformity, optimizing molecular-level charge screening. Radial distribution function analysis demonstrates K^+^’s stronger affinity for SDS head groups, with preferential sulfate coordination reducing surfactant-water hydration interactions. This behavior correlates with hydrogen-bond population reduction, attributed to SDS groups functioning as multidentate ligands—their tetrahedral oxygen arrangement facilitates cooperative hydrogen-bond networks, while counterion-specific charge screening competitively modulates bond formation. The resultant interfacial restructuring enables ordered molecular arrangements with lower system curvature than those observed in Li^+^ and Na^+^-containing systems. These findings elucidate counterion-mediated interfacial modulation mechanisms and establish K^+^ as an optimal candidate for enhancing PFH_X_A/SDS mixture performance through hydration-radius screening. The work provides molecular-level guidelines for designing eco-friendly surfactant systems with tailored interfacial properties.

## 1. Introduction

Over the past several decades, the mixtures of fluorocarbon and hydrocarbon surfactants have been extensively employed in various industrial sectors, including firefighting, detergents, and wetting agents. Among these applications, aqueous film-forming foams (AFFF) stand out as highly effective fire-suppressing agents for combating liquid fires. Fluorosurfactants play a pivotal role in AFFF formulations, where they exert a unique capability to reduce surface tension, thereby enhancing the foam’s fire-extinguishing properties [1]. Consequently, this enhancement has bolstered the surface activity and spreading capabilities of the film-forming foams. However, recent research has uncovered that perfluoroalkyl and polyfluoroalkyl substances (PFAS), a class of synthetic chemicals characterized by their exceptional chemical and thermal stability, also exhibit high bioaccumulative potential and significant aquatic toxicity [2,3]. Awareness of the detrimental and adverse impacts on health and ecology due to the presence of fluorinated surfactants in firefighting foams has significantly heightened in recent years [4]. Due to the presence of perfluorooctanesulfonate (PFOS), a key component of Aqueous Film-Forming Foams (AFFF), the application of these foam-based fire suppressants has been subject to restrictions by the United Nations Environment Programme (UNEP). Utilizing short-chain fluorocarbon surfactants presents a viable alternative. A large body of literature emphasizes that PFHxA, unlike conventional long-chain PFOS, exhibits environmentally benign properties—no significant persistence, bioaccumulation or ecotoxicity. This transition significantly reduces the potential risks these substances pose to both the environment and human health [5,6]. Furthermore, short-chain fluorocarbon surfactants have demonstrated superior performance in the wetting and aggregation mechanisms for the suppression of coal dust, showcasing their efficacy in industrial applications where dust control is paramount [7]. While the transition to short-chain fluorocarbon surfactants addresses environmental concerns, their interfacial performance is intricately linked to molecular-level interactions that remain poorly understood. A critical factor influencing surfactant behavior lies in the role of counterions, which modulate electrostatic interactions and aggregate conformation. The imperative to investigate short-chain fluorocarbon surfactants lies in their capacity to offer safer, more environmentally friendly, and higher-performing alternatives. The benefits of mixed surfactant systems are markedly superior to their individual counterparts. The combination of fluorocarbon and hydrocarbon surfactants, in particular, offers a synergistic blend with remarkable efficacy and broad application prospects in numerous domains [8,9]. These attributes are essential to address the challenges posed by long-chain perfluorinated compounds and to meet contemporary society’s demands for sustainable development. To optimize the performance of these mixed systems, a fundamental understanding of ion-surfactant interactions at interfaces is imperative. In pursuit of a deeper understanding of the influence of counterions on the conformation of surfactant aggregates, recent studies have highlighted this area as a significant research gap. As discovered by Phan, C. M. et al. [10], surfactants and their counter-ions exert contrasting influences on the tension of the air/liquid interfacial layer. Moreover, the impact of counterions on adsorbed monolayers has not yet been systematically explored. This underscores the need for further investigation into how counterions affect the interfacial behavior of surfactants [11].

Traditional experimental techniques often face limitations in resolving molecular-scale phenomena, necessitating advanced computational approaches. Molecular dynamics (MD) simulation, acclaimed as a robust investigative instrument, has been extensively harnessed to delve into the atomic-scale architecture and dynamics of biological and chemical phenomena. This method simulates the motion of atoms or molecules under a prescribed potential energy function by numerically solving Newton’s equations of motion [12], thereby unveiling the intricate choreography of microscopic systems. MD simulation serves as a powerful analytical tool, offering profound insights into the microscopic structure and dynamical behavior of materials. Its broad applications span across various disciplines including physics, chemistry, biology, and materials science [13], facilitating a deeper comprehension of complex systems at the molecular level. In the context of surfactant research, MD simulations have proven particularly valuable for elucidating interfacial adsorption mechanisms. Numerous researchers have employed MD simulation to demystify the structural characteristics and adsorption mechanisms of various surfactants, shedding light on the intricate processes that govern their behavior at interfaces. Gang et al. investigated the characteristics of protonated surfactin monolayers at the air/water interface using molecular dynamics simulations. The study provided detailed insights into the surface parameters and structural attributes of the adsorbed protonated surfactant monolayer [14]. Nan et al. [15] concentrated on the enhancement of oil recovery through MD simulations, offering a detailed molecular-level analysis of agent interactions within oil reservoir environments. In summary, MD simulation is an indispensable tool for investigating the interfacial behavior of surfactants, offering a detailed, molecular-level understanding that complements and extends traditional experimental methods. Its predictive and analytical capabilities make it a cornerstone of modern surfactant research and development.

This study expands on our prior work [16] by conducting a comprehensive molecular dynamics simulation analysis of the short-chain fluorocarbon surfactant PFH_X_A and anionic surfactant SDS at the air/water interface, examining key parameters to reveal their aggregation and micro-interactions. Additionally, we explored the underlying microscopic interaction mechanisms between counterions and surfactants, aiming to provide insights and theoretical foundations for the development and design of novel mixed surfactants.

## 2. Results and Discussion

### 2.1. Stability of Simulated Systems

The root mean square deviation (RMSD) and mean square displacement (MSD) serve as essential quantitative indicators for evaluating the equilibrium state of molecular systems. In the context of this investigation, RMSD and MSD were deployed to scrutinize the temporal progression of conformational fluctuations within surfactant molecules, with both parameters quantifying the mean atomic deviation from their average positions [17,18]. The RMSD and the MSD were rigorously tracked to ascertain that the PFH_X_A/SDS system had achieved a state of dynamic equilibrium.

**Figure 1:**
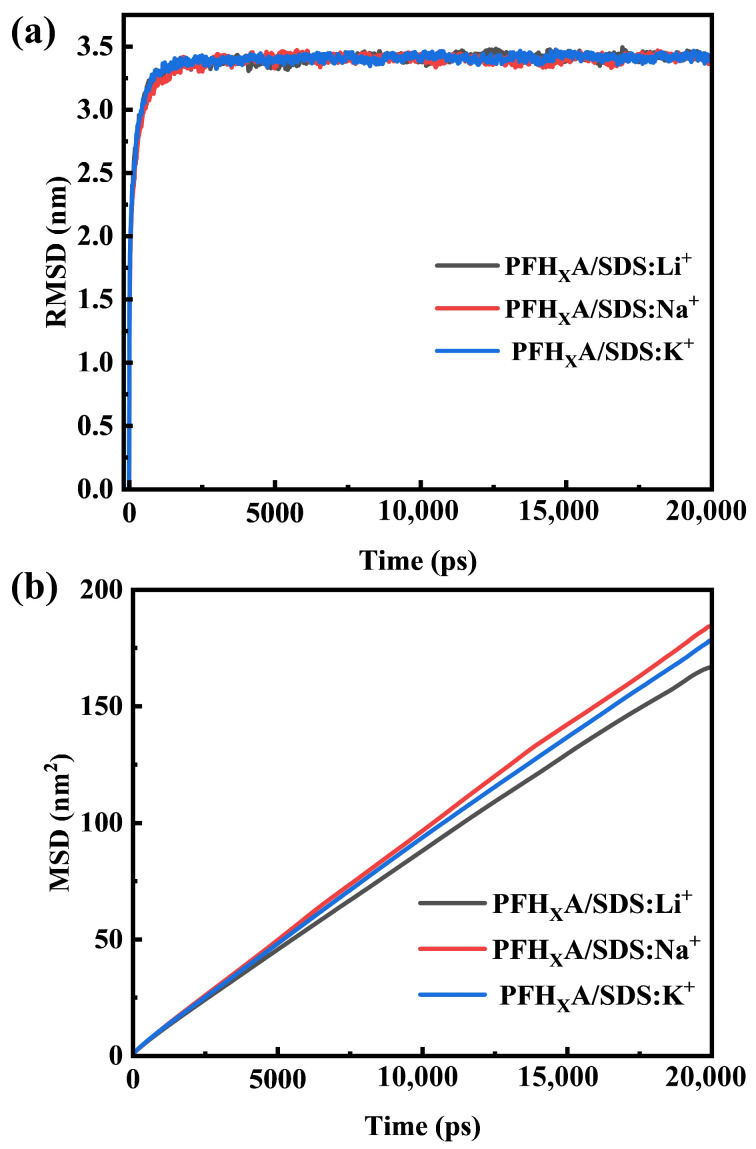
PFH_X_A/SDS with antagonistic ions (**a**) RMSD and (**b**) MSD.

Figure 1 presents a graphical representation of the RMSD and MSD values as a function of simulation time, highlighting the system’s kinetic response upon the commencement of molecular dynamics simulations. An initial peak in RMSD values is observed, followed by a stabilization phase that plateaus after a 5000-picosecond simulation timeframe, signifying the system’s attainment of equilibrium. The average RMSD values for the PFH_X_A/SDS: Li^+^, PFH_X_A/SDS: Na^+^, and PFH_X_A/SDS with K^+^ counterions were determined to be (3.409 ± 0.09) nm, (3.402 ± 0.11) nm, and (3.407 ± 0.07) nm, respectively. Within the MSD analysis, the slope of the plot is directly proportional to the diffusion coefficient, with the stabilization of this coefficient over time indirectly reflecting the system’s stability. These findings not only validate the equilibrium state of the system but also suggest a notable transformation of surfactant molecules at the air–liquid interface within the mixed systems.

The negligible variations in RMSD values and the minimal changes in the MSD slope observed in subsequent simulation intervals further substantiate that the system has reached a stable equilibrium [19,20]. This observed stability is paramount for confirming the reliability and efficacy of the molecular dynamics simulations conducted in this study, ensuring that the insights and conclusions drawn from these simulations are founded upon a robustly equilibrated molecular system.

### 2.2. One-Dimensional -Density Distribution

**Figure 2:**
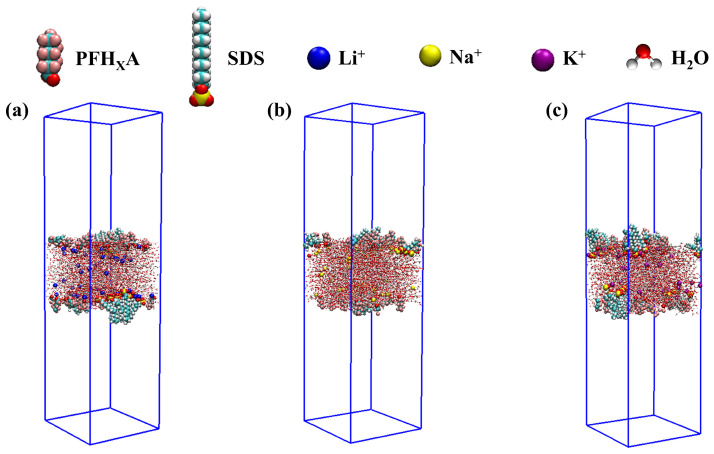
Snapshots of the simulation chamber at the end of the simulation (20 ns) for different surfactant mixing molar ratios: (**a**) PFH_X_A/SDS: Li^+^, (**b**) PFH_X_A/SDS: Na^+^, (**c**) PFH_X_A/SDS: K^+^.

Figure 2 offers a detailed visual representation of the equilibrium monolayer structure that emerges from the PFH_X_A/SDS mixture upon completion of the adsorption process. The lateral perspective provides critical insights, revealing that after an adequately long simulation epoch, the blend of surfactants accumulates precisely at the air/liquid interface. This aggregation results in the formation of a coherent and stable monolayer, which is essential for understanding the interfacial properties of the system. Within this meticulously organized assembly, the hydrophilic head groups of the constituent surfactant molecules are observed to be fully immersed within the aqueous phase, while their hydrophobic tails extend into the aerial expanse, creating a distinct dual-layer arrangement. Notably, a significant concentration of counterions is discerned in the immediate vicinity of these hydrophilic head groups, which possess countervailing charges. This observation underscores a pronounced electrostatic binding affinity between the counterions and the surfactant head groups that reside at the interfacial plane.

**Figure 3:**
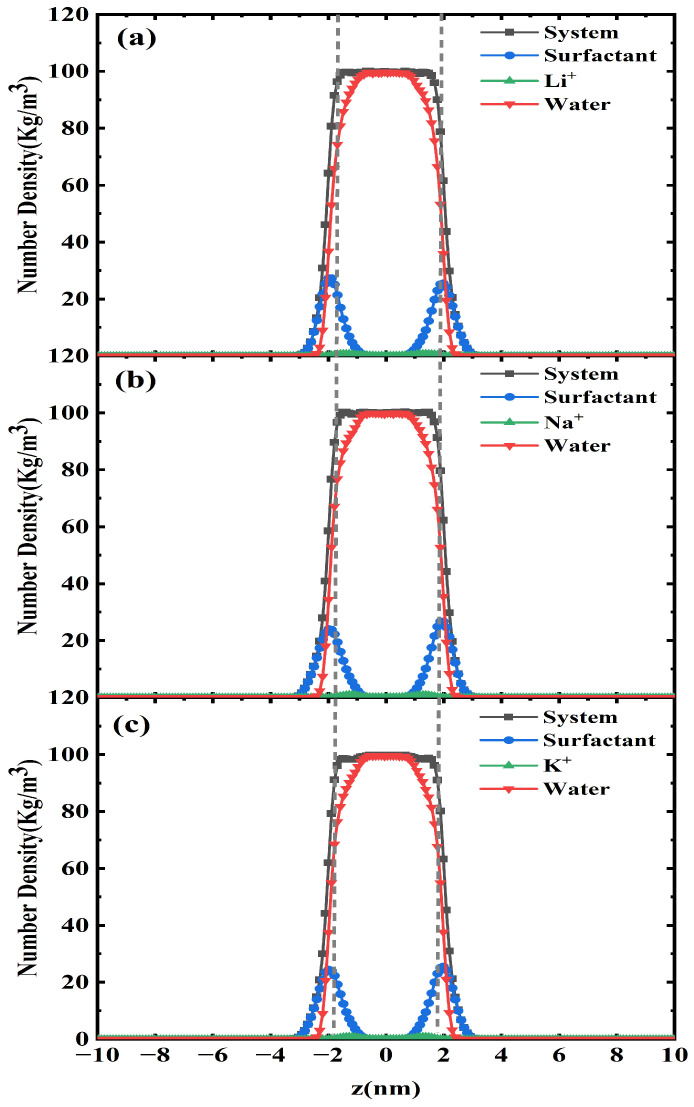
*Z*-axis distribution of number densities: (**a**) PFH_X_A/SDS: Li^+^, (**b**) PFH_X_A/SDS: Na^+^, and (**c**) PFH_X_A/SDS: K^+^.

**Figure 4:**
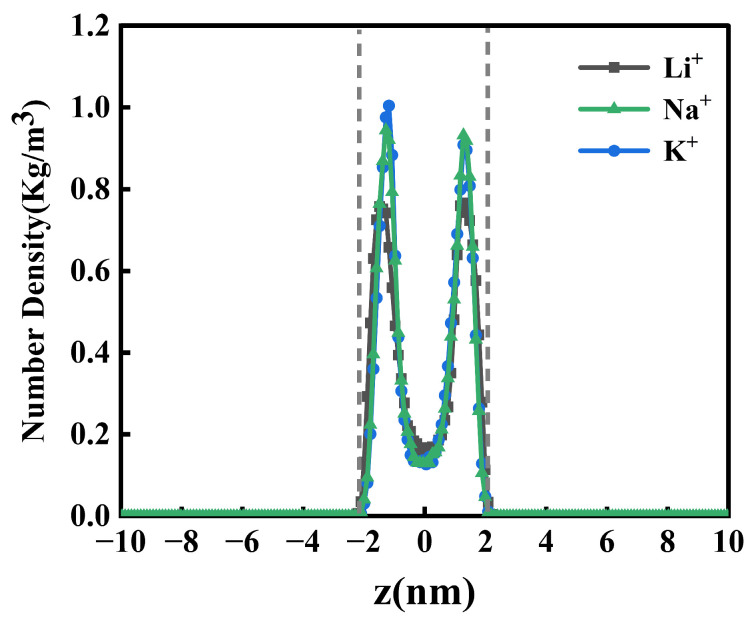
PFH_X_A/SDS: counterbalance ion number density distribution.

To conduct a thorough evaluation of system, we performed spatial mapping of whole system, surfactant molecules, counterions (Li^+^, Na^+^, K^+^), and water molecules across the air/liquid interface using advanced number density profiling along the x-y plane (Figure 3). Despite the apparent similarity in the one-dimensional number density profiles observed under comparable surfactant-to-water ratios and approximately equivalent numbers of water molecules across the Li^+^, Na^+^, and K^+^ systems, quantification of the average number densities reveals subtle variations; specifically, the mean surfactant number densities (kg/m^3^) are 2.547, 2.544, and 2.545 for the Li^+^, Na^+^, and K^+^ systems, respectively, while the corresponding average water number densities (kg/m^3^) are 17.930, 17.916, and 17.917, thereby numerically confirming the inherent differences between the systems illustrated in Figure 3. The coordinate system was established with the simulation box centroid at z = 0 nm. The observed homogeneous distributions of both water and surfactant molecules at 298.15 K confirm interfacial stability, demonstrating thermodynamic equilibrium under specified conditions. The individual detailed number density distribution is shown in Figure S1. For enhanced resolution of counterion-specific behavior, Figure 4 delineates a Hofmeister-type correlation between the peak counterion density and ionic hydration characteristics in the PFH_X_A/SDS system, following the sequence Li^+^ < Na^+^ < K^+^. Potassium ions exhibit superior efficacy in screening electrostatic repulsions between anionic head groups compared to Li^+^ and Na^+^, a phenomenon governed by their distinct hydration thermodynamics rather than bare ionic radius. While K^+^ possesses the largest crystallographic radius (2.04 Å vs. Na^+^: 1.86 Å; Li^+^: 1.52 Å), its reduced hydration radius (3.31 Å vs. Na^+^: 3.58 Å; Li^+^: 3.82 Å) and weaker hydration shell enable a closer approach to the negatively charged –SO_4_^−^ head groups [21]. This physicochemical duality manifests through three synergistic effects: (1) enhanced charge neutralization via increased local charge density at the interface due to smaller hydrated volume; (2) reduced counterion migration through strengthened interactions with hydrophilic head groups; and (3) optimized electrostatic screening per ion that diminishes lateral repulsion forces. The resultant charge screening promotes tighter molecular packing of PFH_X_A and SDS surfactants into low-curvature monolayers, with K^+^ inducing the most compact interfacial assemblies. This ion-specific hierarchy underscores the predominance of hydration thermodynamics over geometric size in dictating surfactant self-organization. The enhanced interfacial architecture originates in the dynamic hydration-dehydration equilibrium of potassium ions (K^+^), which drives significant improvements in the system’s mechanical resilience and interfacial durability.

### 2.3. Two-Dimensional Density Distribution

**Figure 5:**
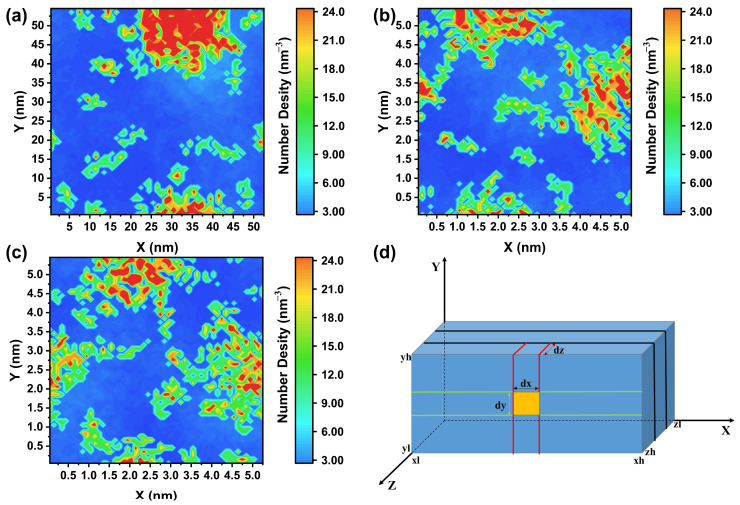
Two-dimensional number density distribution maps of surfactant molecules within the XY plane: (**a**) PFH_X_A/SDS: Li^+^, (**b**) PFH_X_A/SDS: Na^+^, and (**c**) PFH_X_A/SDS: K^+^. (**d**) Illustrative depiction of the variables involved in the two-dimensional numerical density analysis.

To gain insights into the adsorption and aggregation behaviors of mixed surfactants at the air-water interface, we employed TCL [22] scripting within the VMD 1.9.3 version software suite to calculate the two-dimensional number density of surfactant molecules on the XY plane, as depicted in Figure 5. The computational outcomes were derived from Equation (1). The depiction of the variables involved in the two-dimensional numerical density analysis is illustrated in Figure 5d.

(1) $$\Delta V=\left[\frac{\left(zh-zl\right)}{zr}\right]*\left[\frac{\left(xh-xl\right)}{xr}\right]*\left[\frac{\left(yh-yl\right)}{yr}\right]$$

In Equation (1), the parameter Δ*V* defines the cuboid volume, with its dimensions calculated as the span between upper and lower boundaries: ΔZ = *zh* − *zl* (*Z*-axis), ΔX = *xh* − *xl* (*X*-axis), and ΔY = *yh* − *yl* (*Y*-axis). The variables zr, xr, and yr specify the discretization levels (i.e., partitioned intervals) along each respective spatial coordinate.

The color distribution within the two-dimensional number density maps provides a nuanced understanding of the surfactant aggregation at interfaces within various mixed systems, as well as the concept of compactness. In our computational study examining the surfactant number density at the air–liquid interface, we discerned a pronounced variation in the distribution profiles between the PFH_X_A/SDS: Li^+^ system and its Na^+^ and K^+^ analogs. The 2D number density distribution of the PFH_X_A/SDS: Li^+^ system revealed an abundance of deep blue and deep red regions, indicative of a non-uniform surfactant distribution with partial aggregation occurring at the gas–liquid interface. In the two-dimensional number density distribution of the PFH_X_A/SDS: K^+^ system within the XY plane, an increased presence of green regions is observed, corresponding to a reduction in the deep red and deep blue areas. This observation indicates a more uniform distribution of surfactant molecules at the air–liquid interface within the system. The distinct distribution patterns observed for the PFH_X_A/SDS: Li^+^, Na^+^, and K^+^ systems underscore the significant influence of the counterion’s nature on the interfacial arrangement of surfactant molecules. This finding is further corroborated by the 1D number density analysis and the final conformations simulated for the different systems as depicted in Figure 2, which may reveal the nuanced interactions and arrangements of surfactant molecules in response to the varying chemical environments provided by the different counterions.

### 2.4. Surface Tension Calculation

Surface tension measurements are recognized as quintessential parameters in the quantitative appraisal of surfactant interfacial characteristics, serving as pivotal benchmarks for gauging their efficacy at the interface [23]. By employing Equation (2), we meticulously calculated the surface tension and sample standard deviation across three distinct experimental systems. As meticulously delineated in Table 1, a discernible and systematic trend of decreasing surface tension with an increment in the radius of the counterion was observed, which strictly adhered to the hierarchical order of PFH_X_A/SDS: Li^+^ > PFH_X_A/SDS: Na^+^ > PFH_X_A/SDS: K^+^. This observed gradient not only underscores but also vividly illustrates an escalation in the binding affinity between the counterions and the composite surfactant head groups, progressively advancing from the smallest Li^+^ to the largest K^+^.

Such an increase in binding strength is intricately correlated with an enhancement in the molecular structural stability [24] of the surfactant system, which in turn is directly associated with a concomitant and significant reduction in surface tension. Notably, within the PFH_X_A/SDS system that was integrated with K^+^, the formation of the densest surfactant monolayer at the interface was meticulously observed, coupled with the lowest recorded surface tension among the systems studied. This observation not only exemplifies but also underscores the most pronounced impact of K^+^ on the aggregation dynamics occurring at the PFH_X_A/SDS air–liquid interface, thereby highlighting the pivotal role of counterion radius in modulating the interfacial behavior of surfactants.

(2) $$\gamma =\frac{1}{2}L_{z}\left[p_{zz}-\frac{p_{xx}+p_{\mathrm{yy}}}{2}\right]$$

*p _zz_* is the normal stress, and *p _xx_* and *p _yy_* are the tangential stresses. *L_z_* denotes the length along the *z*-axis normal to the air/liquid interface. The term 1/2 accounts for the dual interfaces in the simulation.

### 2.5. Radial Distribution Function

The hydrodynamic behavior in the vicinity of the hydrophilic head groups of surfactants plays a pivotal role in dictating the behavior of surfactants in both solution and at interfaces. This behavior can be quantified and elucidated through the analysis of the radial distribution function (RDF), a powerful tool that not only reflects the microstructural characteristics of the particles but also reveals the intensity of their interactions [25]. By examining the RDF, we can investigate the spatial distribution of water molecules at the interface and their arrangement around different surfactant head groups within a mixture. The positions of the peaks in the RDF reflect the distances between the two reference species. Smaller peak positions indicate closer proximity and more intimate interactions between the two reference species. Meanwhile, the peak intensities of the RDF represent the number of reference species B at a specific distance from reference species A. Stronger peak intensities suggest a greater number of reference species B at that distance, implying more robust interactions.

**Figure 6:**
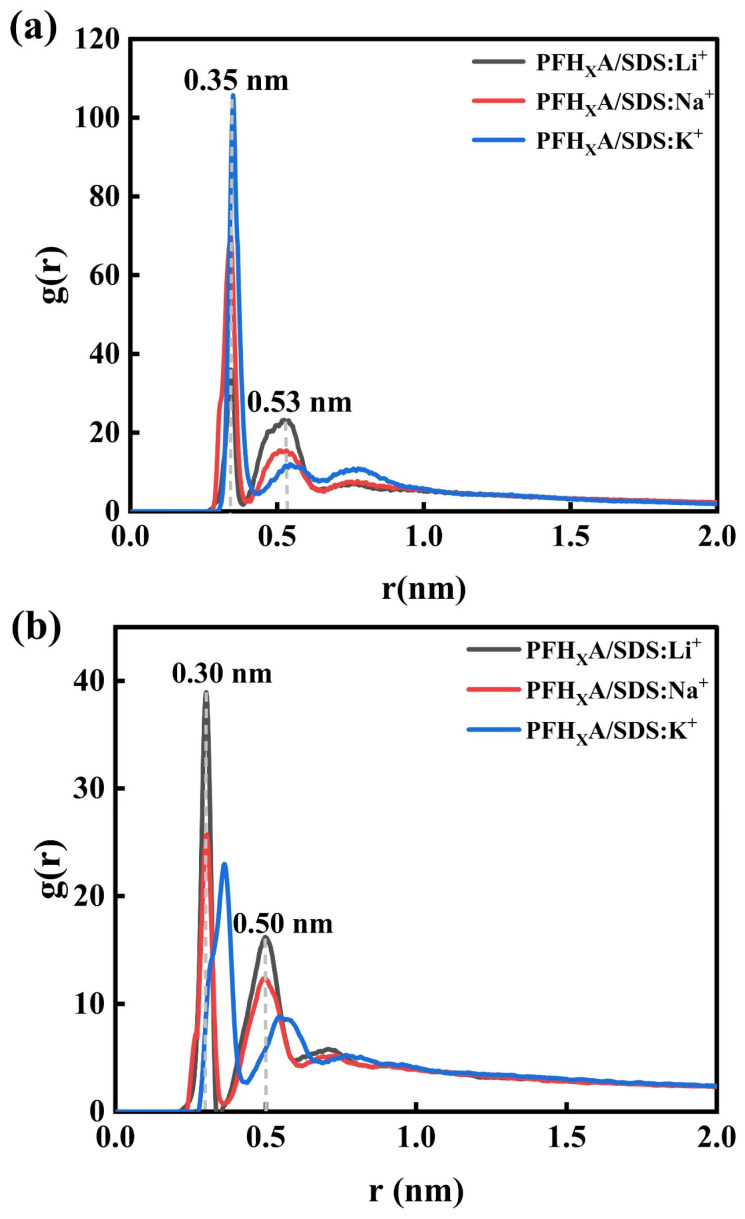

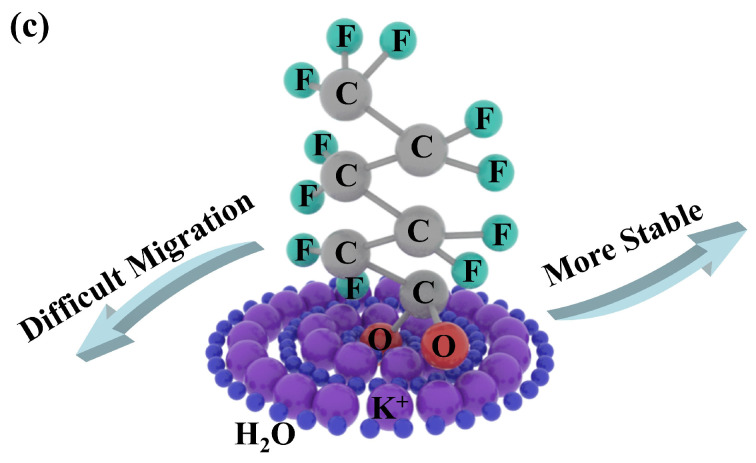
Radial distribution function between (**a**) SDS head groups and (**b**) PFH_X_A head groups with different counterbalance ions in surfactant complex systems. (**c**) Schematic diagram of the RDF with PFH_X_A as a column.

To quantify the impact of counterions on the interactions between surfactants and water, the RDF between the head groups of the PFH_X_A/SDS mixture and counterions were computed, as illustrated in Figure 6. Each peak in the RDF represents a counterion layer, with peak heights indicating the distribution intensity at specific locations. Counterions bind to the head groups of PFH_X_A and SDS due to electrostatic interactions, and the positions of the RDF peaks suggest penetration into the primary and secondary hydration shells. The weak intensity of g(r) [26] confirms the shielding effect of counterions on the interactions between head groups and water molecules, disrupting the original water orientation around the polar heads and altering the hydrogen bond structure [27]. The varying peak intensities across the RDF indicate that the size of the counterion radius significantly influences the water structure around the PFH_X_A and SDS interface and also highlight the substantial impact of strong interactions between Li^+^, Na^+^, and K^+^ and the surfactant on their aggregation at the air/liquid interface.

Figure 6a presents the radial distribution function (RDF) profiles for the interaction between SDS head groups (–SO_4_^−^) and counterions (Li^+^, Na^+^, K^+^). A well-defined primary counterion shell is observed at 0.35 nm, with peak intensities exhibiting a distinct cation ordering (K^+^ > Na^+^ > Li^+^). Notably, K^+^ displays the highest RDF maximum (105.87), reflecting its superior coordination within the primary solvation shell. This enhanced affinity is attributed to K^+^’s smaller hydration radius (3.31 Å vs. Na^+^: 3.58 Å; Li^+^: 3.82 Å) and weaker hydration shell, which allows closer proximity to the anionic head group [28]. The reduced hydration strength diminishes counterion mobility and strengthens electrostatic interactions, thereby enhancing charge shielding effects. This improved electrostatic screening mitigates lateral repulsion between PFH_X_A and SDS molecules, promoting tighter molecular packing into low-curvature monolayers [29]. A secondary counterion shell with reduced structural intensity appears at 0.53 nm, and the RDF curves plateau beyond 0.8 nm, indicating well-defined solvation shells. Comparative analysis reveals that K^+^ exhibits optimized binding thermodynamics with sulfonate moieties compared to Li^+^ (RDFmax = 36.10) and Na^+^ (RDFmax = 70.69).

In contrast, Figure 6b illustrates the carboxylate counterion (–COO^−^) RDF profiles for PFHXA/SDS systems. The primary peak intensities at 0.30 nm decrease in the order Li^+^ > Na^+^, with the PFH_X_A/SDS: K^+^ system maintaining its first counterion peak at 0.35 nm but exhibiting a reduced RDF maximum of 22.75. This contrasts sharply with the SDS head group (–SO_4_^−^), which demonstrates a significantly higher RDF maximum (105.87) with K^+^ counterions compared to the 39.11 observed for PFH_X_A head group (–COO^−^) interactions with Li^+^. This pronounced disparity in coordination strength indicates markedly weaker binding affinity between Li^+^ and the carboxylate head groups relative to the robust K^+^-sulfate interactions in the PFH_X_A/SDS mixed system.

### 2.6. Electrostatic Potential on Van Der Waals Surfaces

**Figure 7:**
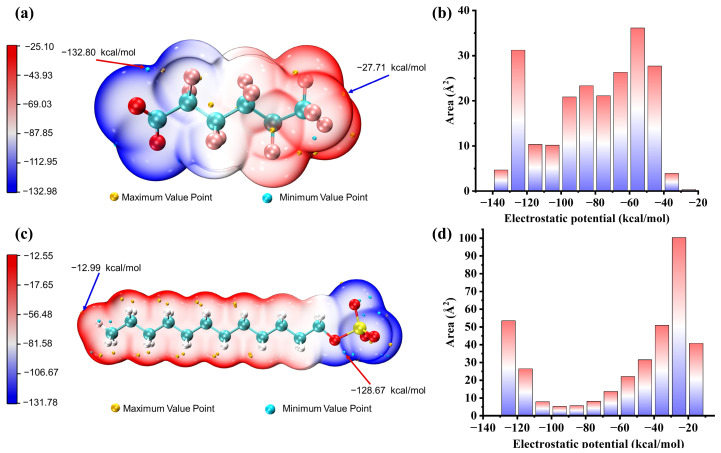
Analysis of molecular electrostatic potential extremes and surface area distributions on van der Waals surfaces: PFH_X_A (**a**,**b**) and SDS (**c**,**d**).

The analysis of the electrostatic potential (ESP) distribution on PFH_X_A and SDS surfaces reveals critical cooperative mechanisms between anionic surfactants. Following geometry optimization at the B3LYP/6-31G (d, p) level (Gaussian 09 revision A.02), quantitative molecular surface electrostatic potential (ESP) analysis performed through Multiwfn’s wavefunction processing core—coupled with VMD v1.9.8′s Tachyon-rendered volumetric visualization—reveals atomistically resolved surface polarization patterns across van der Waals boundaries, demonstrating synergistic integration with independent reduced density gradient (RDG) non-covalent interaction analysis [30,31] (Figure 7). Detailed information can be found in Tables S1–S4.

For PFH_X_A, the global minimum ESP value (−132.80 kcal/mol) corresponds to the carboxylate oxygen atoms (–COO^−^) in the head group, reflecting strong localized negative charge density, while the global maximum ESP value (−27.71 kcal/mol) is attributed to the fluorinated methyl atoms in the tail group. In panel Figure 7b, surface regions with ESP values below −130 kcal/mol (head group) and above −30 kcal/mol (tail) occupy minimal vdW surface areas. The uniform ESP distribution, driven by the short fluorocarbon chain, underpins PFH_X_A’s role in interfacial assembly via homogeneous charge dispersion. Conversely, SDS exhibits a comparable head group (PFH_X_A) electronegativity (–SO_4_^−^ ESP minimum: −128.67 kcal/mol) but with spatially dispersed and concentrated charge distribution. The global maximum ESP value (−12.99 kcal/mol) is localized at the hydrogen atoms in the dodecyl tail group. As illustrated in panel Figure 7d, surface regions with ESP values ranging from −120 to −80 kcal/mol occupy substantial areas corresponding to sulfonate group interactions, creating localized high-affinity sites for counterion binding. The most extensive surface regions (ESP values: −40 to −10 kcal/mol) correlate with the dodecyl chain structure, indicating hydrophobic interactions significantly contribute to molecular assembly. Unlike PFH_X_A’s uniform distribution, SDS exhibits distinct electrostatic characteristics, featuring a concentrated yet dispersed charge profile.

The synergistic behavior between PFH_X_A and SDS arises from structural–electrostatic complementarity: SDS’s extended dodecyl chain enhances hydrophobic interactions to compensate for PFH_X_A’s shorter fluorocarbon tail, optimizing micellar stability. Simultaneously, PFH_X_A’s uniform negative charge distribution counterbalances SDS’s concentrated yet dispersed charge profile, establishing an electrostatic gradient that promotes cooperative self-assembly. This interplay between PFH_X_A’s head group-dominated electrostatics and SDS’s hybrid hydrophobic/electrostatic domains enables balanced molecular aggregation through dual mechanisms—electrostatic interactions between charged regions and sterically optimized packing geometry. These findings highlight how tailored ESP distributions govern surfactant synergism in interfacial systems.

### 2.7. Number of Hydrogen Bonds

**Figure 8:**
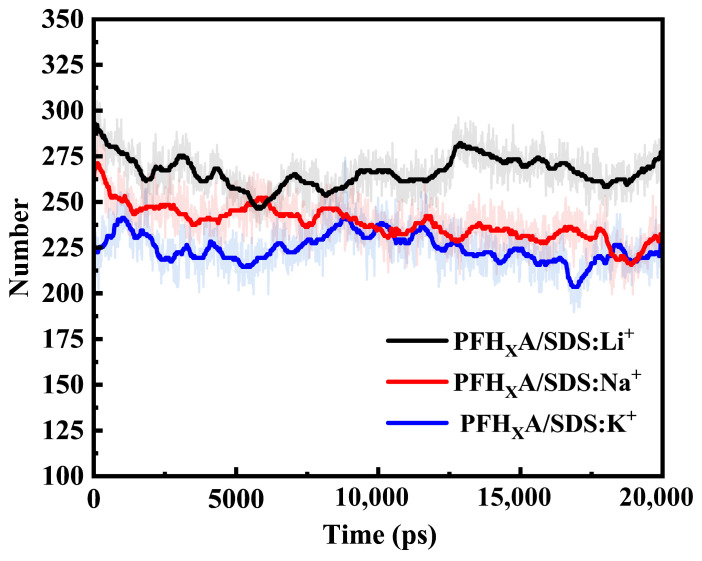
Number of hydrogen bonds for surfactant molecules in different systems over simulation time.

In order to gain a deeper understanding of the interactions between surfactant mixtures with different counterions and water molecules, we have conducted a computational analysis of the hydrogen bonding between these systems and water. The criterion for hydrogen bond determination is as follows: The –H…acceptor distance must not exceed 0.35 nm, while the donor–H…acceptor angle deviation from linearity should not exceed 30 degrees [32,33]. The calculated hydrogen bonding results are shown in Figure 8.

Figure 8 demonstrates significant variations in hydrogen bond formation across the studied systems. A clear positive correlation emerges between counterion hydration radius and hydrogen bond population, with the PFH_X_A/SDS: K^+^ system exhibiting substantially fewer hydrogen bonds than the PFH_X_A/SDS: Li^+^ counterpart. In the PFH_X_A/SDS mixed system, the SDS surfactant dominates hydrogen bond formation through its sulfate head group (–SO_4_^−^). This group functions as both an efficient hydrogen-bond acceptor and a multidentate ligand due to two key characteristics. (1) The electronegative oxygen atoms, particularly those carrying partial negative charges, effectively engage proton donors (e.g., water molecules) through lone pair interactions. (2) The tetrahedral geometry of SDS head group (–SO_4_^−^) provides four spatially accessible oxygen atoms capable of simultaneous coordination with multiple hydrogen-bond donors. This structural configuration enhances hydrogen-bond cooperativity, thereby reinforcing hydration stability.

Section 2.5 reveals that the peak order for counterion (Li^+^, Na^+^, K^+^) association with SDS head groups directly correlates with observed hydrogen bond quantities. The dominant electrostatic interactions between counterions and SDS head groups exceed water-mediated hydrogen bonding effects. Furthermore, counterions induce steric hindrance at potential hydrogen-bonding sites. These findings establish counterion selection as an effective strategy for systematically regulating hydrogen bond populations to engineer specific mixed surfactant properties.

### 2.8. Solvent-Accessible Surface Area

The area occupied by the hydrophilic moiety corresponds to the spatial domain delineated by the hydrophilic components within the surfactant molecule. Comprehending the interfacial characteristics of surfactant molecules is paramount for dissecting the nuances of their structural evolution within solvent matrices. The solvent-accessible surface area [34] (SASA) of the hydrophilic moieties serves as a metric for the area engaged in solvent contact, thereby mirroring the surfactant’s interfacial profile at the air–liquid boundary. Delving into the SASA of these hydrophilic groups is instrumental in garnering a deeper comprehension of the simulation system’s dynamics.

**Figure 9:**
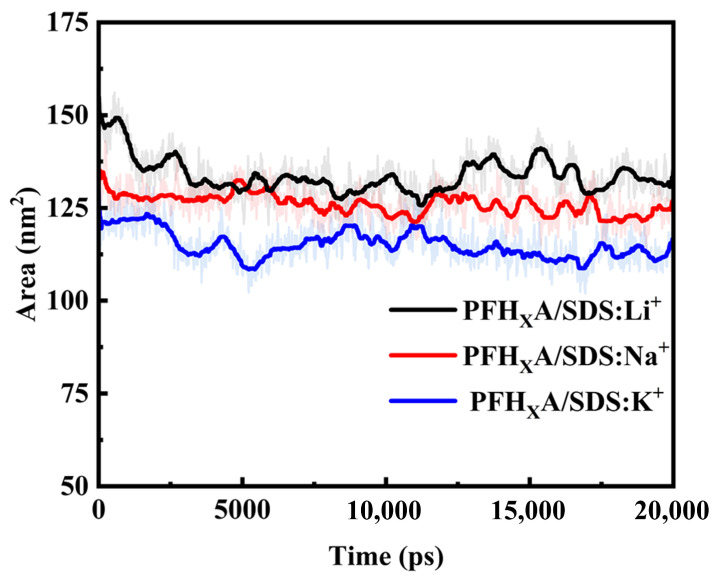
The variation curve of solvent-accessible surface area (SASA) for surfactant molecules in different systems over simulation time.

This section delineates the computational assessment of solvent-accessible surface area (SASA) across three distinct PFH_X_A/SDS systems with different counterions (Li^+^, Na^+^, K^+^), as represented in Figure 9. We observe a positive correlation between SASA and counterion hydration radius, with the K^+^ system exhibiting the most reduced SASA. This reduction primarily stems from thermodynamic factors. Decreased counterion hydration radius increases counterion concentration near surfactant head groups, diminishing electrostatic repulsion and promoting tighter, more organized interfacial assembly [35]. Crucially, this thermodynamic driving force is kinetically facilitated in K^+^ systems, as evidenced by uniform surfactant distributions in 2D density distribution (Section 2.3) indicating efficient molecular reorganization dynamics. This kinetic advantage enables more ready access to the thermodynamically favored low-SASA state compared to Li^+^ systems. The resultant augmented compactness collectively enhances surfactants’ capacity to diminish surface tension, corroborating experimental data in Section 2.4.

## 3. Modeling and Simulation Details

### 3.1. Modeling

**Figure 10:**
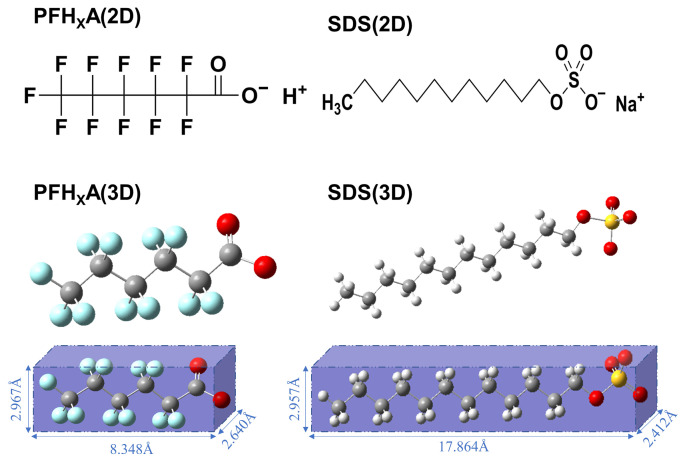
Molecular structure 2D and 3D of PFH_X_A and SDS.

In our preceding research endeavors, we discerned that the most optimal outcomes were achieved when PFH_X_A and SDS surfactants, in various molar ratios, were combined at an equimolar ratio, with sodium ions (Na^+^) as the counterions. This study employs a 1:1 molar ratio of PFH_X_A to SDS—previously established as the optimal surfactant composition for interfacial performance—as the foundational model system to elucidate the impact of diverse counterions on interfacial behavior, thereby enabling further optimization of the surfactant complex. The 2D and 3D structural representations of PFH_X_A (a) and SDS (b) are depicted in Figure 10, showcasing the molecular architecture essential for our investigation. In this case, the volume of the surfactant is related to the surface tension [36], and the detailed parameters are shown in Figure 10. Utilizing GaussView 5.0 version software, we initially constructed the molecular structures of these surfactants. Subsequently, we performed geometric optimization employing the B3LYP/6-31G (d, p) level of theory with Gaussian 09 revision A.02 software [37] to (i) determine initial lowest-energy molecular geometries under vacuum conditions and (ii) generate electrostatic potential (ESP)-derived atomic charges for classical force field assignment.

**Figure 11:**
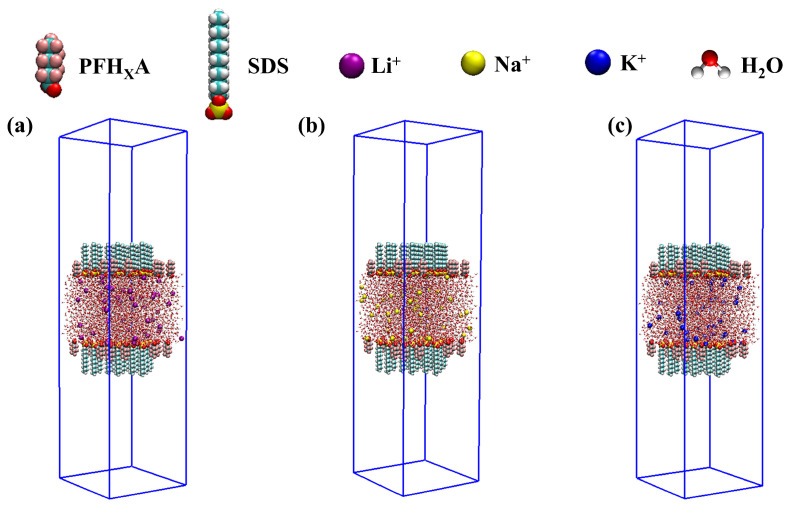
The initial model of the system: (**a**) PFH_X_A/SDS: Li^+^, (**b**) PFH_X_A/SDS: Na^+^, (**c**) PFH_X_A/SDS: K^+^.

The foundational model of our simulated system, as illustrated in Figure 11, is composed of a computational box with dimensions of 5 nm by 5 nm by 20 nm. To demonstrate the appropriateness of our simulation box dimensions, the extended simulation box Z-dimension from 20 nm to 25 nm for the K^+^-containing PFH_X_A/SDS system yielded surface tensions of 20.9 mN/m and 21.3 mN/m, respectively. See Figure S5 for details. This box was meticulously filled with a central layer of water, 5 nm in thickness, using software directives, with surfactant molecules PFH_X_A and SDS randomly and uniformly dispersed at the water interface in a 1:1 ratio. The detailed schematic top and front views of the system are provided in Figures S2 and S3. The system was designed to emulate an air/surfactant/water/surfactant/air interfacial configuration. To minimize potential errors arising from the initial random configuration, new initial models were regenerated (Figure S4), which demonstrated negligible effects on the simulated surface tension values. The construction of the initial structure was facilitated by the PACKMOL software (version 20.2) [38] in conjunction with solvate and gmx editconf tools within the GROMACS 2018.8 version software suite [39,40], ensuring consistency across all simulated conditions. Following this, discrete quantities of assorted counterions (Li^+^, Na^+^, K^+^) were meticulously incorporated to preserve electrical neutrality within the system. The centroid of the rectangular simulation box coincides with the system’s center of mass, ensuring symmetrical alignment and uniformity across the upper and lower interfacial regions. The VMD (version 1.9.8) [41] software was subsequently employed for the manipulation and visualization of dynamic trajectories and snapshot imagery. A comprehensive tabulation of the surfactant molecules, water molecules, and counterions present in the simulation systems is provided in Table 2. The total concentration of surfactant (${\left[\mathrm{surfanctant}\right]}_{tot}$) molecules was calculated to be 0.7392 mol/L using Equation (3) [42], exceeding the critical micelle concentration (CMC).

(3) $${\left[\mathrm{surfanctant}\right]}_{tot}=(\frac{N_{bulk}+N_{surface}}{N_{A}})\;\times \frac{1}{V},$$

where $N_{bulk}$ represents the number of surfactant molecules dissolved in the liquid phase, $N_{surface}$ denotes the number of surfactant molecules residing at the surface phase, $N_{A}$ corresponds to Avogadro’s constant, and *V* is the volume of the liquid phase.

### 3.2. Simulation Details

**Figure 12:**
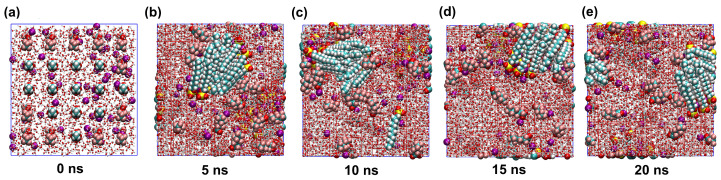
Representative structural reorganization over time in the PFH_X_A/SDS: K^+^ system (**a**–**e**): 0 ns to 20 ns.

To ensure consistency in simulation procedures, GROMACS 2018.8 software package was utilized for all simulations conducted. All simulations employed the CHARMM36 force field—a well-established parameterization for modeling surfactant behavior at interfaces—ensuring methodological consistency with prior investigations in this domain [43], with water molecules modeled according to the SPC/E protocol. The model accurately predicts water properties, especially density and diffusion constants [44]. Periodic boundary conditions were uniformly imposed across the xyz axes in the simulation. The simulated density (998.523 kg/m^3^) and diffusion coefficient (2.42 * 10^−5^ cm^2^/s) demonstrate close agreement with experimental values (997.05 kg/m^3^ and 2.29 * 10^−5^ cm^2^/s, respectively)—confirming force field transferability to aqueous systems. Topology files were generated using the sobtop (version 1.0) software [45]. A tool of generating forcefield parameters and GROMACS topology file. The molecular structure of the surfactants was modeled using a full-atom representation, surfactants with atomic charges determined by Multiwfn [46]. The electronic distribution of surfactant ions is presented in Table 3 and Table 4. Initial energy minimization (EM) of the assembled system was performed using the steepest descent algorithm to (i) resolve steric clashes from initial construction and (ii) relax local strains, followed by conjugate gradient refinement with a maximum force tolerance of 1000 kJ·mol^−1^·nm^−1^ to prevent simulation instability. The simulation was executed with a 2 fs timestep and a total of 10,000 steps. Temperature control was achieved using the velocity-rescaling [47] thermostat. Long-range electrostatic interactions were handled with the Particle Mesh Ewald (PME) [48] algorithm, while a 0.14 nm cutoff was applied for short-range interactions. The LINCS [49] algorithm was employed to constrain bond lengths, with constraint_algorithm = lincs and constraints = h-bonds explicitly applied to all bonds involving hydrogen atoms. The LINCS parameters were set to lincs_iter = 1 (number of correction iterations) and lincs_order = 4 (expansion order of the matrix inversion) to ensure numerical stability. To mitigate size effects, periodic boundary conditions (pbc = xyz) were implemented in all three Cartesian directions of the simulation system. The non-bonded Lennard–Jones potential was truncated at a cutoff distance of 1.4 nm. Subsequently, the system was subjected to a 1 ns equilibration phase within the NPT ensemble, maintaining a pressure of 1 bar with a compressibility of 4.5 × 10^−5^ bar^−1^ using a semiisotropic Berendsen barostat controlling the *Z*-axis, following energy minimization to ensure the stability of the subsequent phase simulation. Numerous studies have conducted simulations of the production phase, spanning durations of 4 to 20 ns [50,51,52]. The simulation was finalized with a 20 ns isothermal isovolumetric (NVT) ensemble to generate phase simulation data, with the concluding 10 ns extracted for analysis. For the representative structural reorganization over time in the PFH_X_A/SDS: K^+^ system, see Figure 12.

## 4. Conclusions

The critical influence of counterion characteristics on surfactant behavior has been extensively demonstrated in previous experimental studies addressing foaming, micellization, and monolayer assembly [53,54,55]. Through systematic molecular simulations, we provided atomistic-level insights into interionic interactions that expanded upon earlier computational investigations [16], while refining our understanding of counterion-mediated effects in surfactant systems.

Key observations from RMSD and MSD analyses confirmed system equilibration prior to data collection. Subsequent evaluation of 1D/2D density profiles established a distinct correlation between ionic radius and counterion localization, with potassium ions (K^+^) inducing the most compact monolayer formation. Comparative analysis revealed K^+^’s markedly more pronounced impact on PFH_X_A/SDS structural organization compared to Li^+^ and Na^+^ counterparts. Surface tension measurements, SASA quantification, and hydrogen-bond statistics consistently identified K^+^-containing systems as exhibiting minimal values across all three parameters. Notably, hydrogen-bonding patterns and SASA distributions demonstrated direct proportionality between hydrated counterion radius and both hydrogen-bond frequency/SASA occupations. This phenomenon originates from the tetrahedrally arranged electronegative oxygen atoms in SDS head groups, which act as multidentate ligands facilitating cooperative hydrogen-bond networks. Counterions with smaller hydration radii (e.g., K^+^) achieved enhanced head group charge screening through closer interfacial accumulation, effectively reducing inter-surfactant electrostatic repulsion and enabling tighter molecular packing. Complementary electrostatic potential analysis revealed synergistic structural–electrostatic matching between PFH_X_A’s uniform charge distribution and SDS’s spatially separated hydrophobic/charged domains. RDF analysis further elucidated how counterion hydration characteristics regulate surfactant-water interactions, with K^+^ demonstrating optimal coordination behavior attributable to its reduced hydration radius and weaker hydration shell. This enhanced counterion binding not only minimizes lateral repulsion between PFH_X_A and SDS molecules but also disrupts water orientation near polar head groups—both critical factors promoting low-curvature monolayer formation.

These collective findings establish that K^+^-modified PFH_X_A/SDS systems achieve superior interfacial organization through optimized counterion-mediated effects, emphasizing the critical importance of hydration characteristics in surfactant mixture design. While molecular dynamics applications in counterion interaction studies remain relatively limited [56], our work advances interfacial understanding in mixed short-chain perfluorinated/hydrocarbon surfactant systems. Future studies will systematically investigate ionic radius and charge valence effects to comprehensively elucidate counterion influences on surfactant interfacial behavior. The revealed structure–property relationships provide fundamental guidelines for developing optimized surfactant formulations through rational counterion selection, which can more comprehensively inform experimental or industrial applications.

## Figures and Tables

**Table 1:** Values of interfacial tension of three surfactant systems.

System	Surface Tension (mN/m)			Stand Deviations
PFH_X_A/SDS: Li^+^	25.8	25.4	25.3	0.2646
PFH_X_A/SDS: Na^+^	21.4	21.9	22.3	0.4848
PFH_X_A/SDS: K^+^	20.9	20.1	20.4	0.4041

**Table 2:** Number of molecules in the simulation for different systems.

System	Box Dimension	Counterions and Quantities	Water Molecule
PFH_X_A/SDS: Li^+^	5 × 5 × 20 (nm)	Li^+^ (50)	3632
PFH_X_A/SDS: Na^+^	5 × 5 × 20 (nm)	Na^+^ (50)	3632
PFH_X_A/SDS: K^+^	5 × 5 × 20 (nm)	K^+^ (50)	3632

**Table 3:** Surfactant PFH_X_A ionic charge distribution.

PFH_X_A					
Atom	Charge	Mass	Atom	Charge	Mass
C1	0.659382	12.01074	F11	−0.20921	18.9984
C2	0.208681	12.01074	F12	−0.19698	18.9984
C3	0.415	12.01074	F13	−0.18355	18.9984
C4	0.289425	12.01074	F14	−0.20478	18.9984
C5	0.33657	12.01074	F15	−0.25376	18.9984
F6	−0.21045	18.9984	F16	−0.24683	18.9984
F7	−0.21001	18.9984	C17	0.744484	12.01074
F8	−0.20967	18.9984	O18	−0.70875	15.99941
F9	−0.15571	18.9984	O19	−0.69536	15.99941
F10	−0.16849	18.9984			

**Table 4:** Surfactant SDS ionic charge distribution.

SDS					
Atom	Charge	Mass	Atom	Charge	Mass
C1	−0.25072	12.01074	H22	−0.00092	1.007941
H2	0.058142	1.007941	C23	0.02459	12.01074
H3	0.058142	1.007941	H24	−0.00912	1.007941
H4	0.058142	1.007941	H25	−0.00912	1.007941
C5	0.117359	12.01074	C26	0.072927	12.01074
H6	−0.01503	1.007941	H27	−0.02215	1.007941
H7	−0.01503	1.007941	H28	−0.02215	1.007941
C8	−0.00952	12.01074	C29	−0.05769	12.01074
H9	0.001923	1.007941	H30	−0.00233	1.007941
H10	0.001923	1.007941	H31	−0.00233	1.007941
C11	−0.03489	12.01074	C32	0.002969	12.01074
H12	−0.00105	1.007941	H33	0.007111	1.007941
H13	−0.00105	1.007941	H34	0.007111	1.007941
C14	0.058223	12.01074	C35	0.343935	12.01074
H15	−0.01667	1.007941	H36	−0.02482	1.007941
H16	−0.01667	1.007941	H37	−0.02482	1.007941
C17	0.021417	12.01074	O38	−0.54093	15.99941
H18	−0.00876	1.007941	S39	1.01068	32.06479
H19	−0.00876	1.007941	O40	−0.58017	15.99941
C20	−0.02905	12.01074	O41	−0.58017	15.99941
H21	−0.00092	1.007941	O42	−0.55972	15.99941

## Data Availability

The original contributions presented in this study are included in the article/supplementary material. Further inquiries can be directed to the corresponding authors.

## Supplementary Materials

The following supporting information can be downloaded at https://www.mdpi.com/article/10.3390/molecules30122592/s1: Figure S1: *Z*-axis Number Density Profiles of PFH_X_A/SDS Mixed Surfactant Systems and Counterion Distributions: (**a**–**c**) PFH_X_A/SDS Ratios of Li^+^, Na^+^, K^+^; Figure S2: The top views initial model of the system: (**a**–**c**) PFH_X_A/SDS Ratios of Li^+^, Na^+^, K^+^; Figure S3: The front views initial model of the system: (**a**–**c**) PFH_X_A/SDS Ratios of Li^+^, Na^+^, K^+^; Figure S4: The top view initial model of the recreate system: (**a**–**c**) PFH_X_A/SDS Ratios of Li^+^, Na^+^, K^+^; Figure S5: Initial System Model (Z-Axis Dimension of 20 nm) (**a**) and Enlarged System Model (Z-Axis Dimension of 25 nm) (**b**): PFH_X_A/SDS Ratios of K^+^; Table S1: RESP Maximum Distribution of Surfactant PFH_X_A Ions; Table S2: RESP Minimum Distribution of Surfactant PFBS Ions; Table S3: RESP Maximum Distribution of Surfactant SDS Ions; Table S4: RESP Minimum Distribution of Surfactant SDS Ions; Detailed parameters of topology files: SDS.itp; PFHXA.itp.
