# Supplementary material for: Counterion-Mediated Assembly of Fluorocarbon–Hydrocarbon Surfactant Mixtures at the Air–Liquid Interface: A Molecular Dynamics Study
Source: Molecules. 2025 Jun 14;30(12):2592. doi: 10.3390/molecules30122592 (PMC12196147; doi:10.3390/molecules30122592)
Supplement: Supplementary file 1 [file molecules-30-02592-s001.zip › molecules-3685792-supplementary.pdf]

Supporting Information

Counterion-Mediated Assembly of Fluorocarbon–Hydrocarbon Surfactant Mixtures at the Air-Liquid Interface: A Molecular Dynamics Study

Xiaolong Quan,† Tong Tong,† Tao Li, Dawei Han, Baolong Cui, Jing Xiong, Zekai Cui, Hao Guo, Jinqing Jiao,* Yuechang Wei,*

State Key Laboratory of Heavy Oil Processing, China University of Petroleum (Beijing), Beijing 102249, China

* Corresponding author. Email address: jiaojq.qday@sinopec.com; weiyc@cup.edu.cn

† These authors contributed equally to this work.

1D-Density

(a)

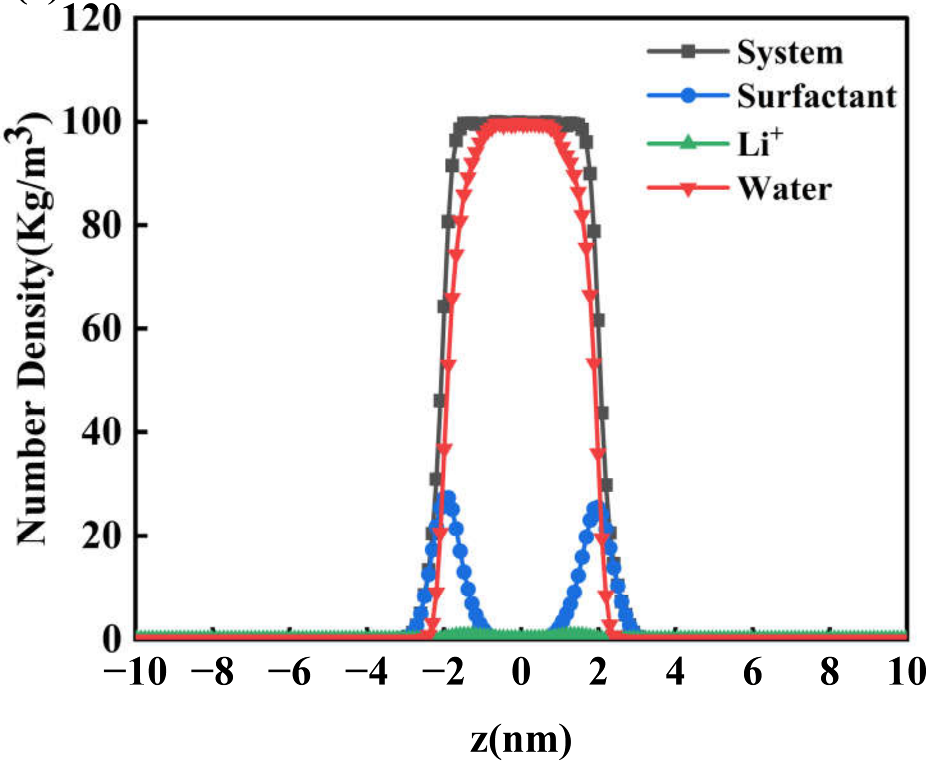

(b)

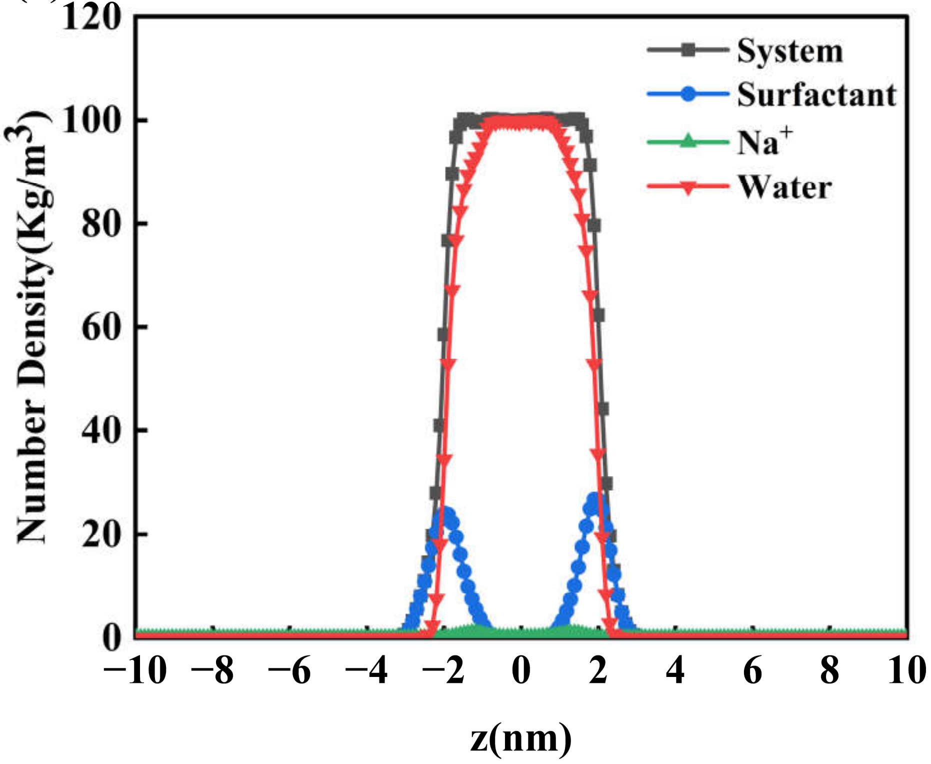

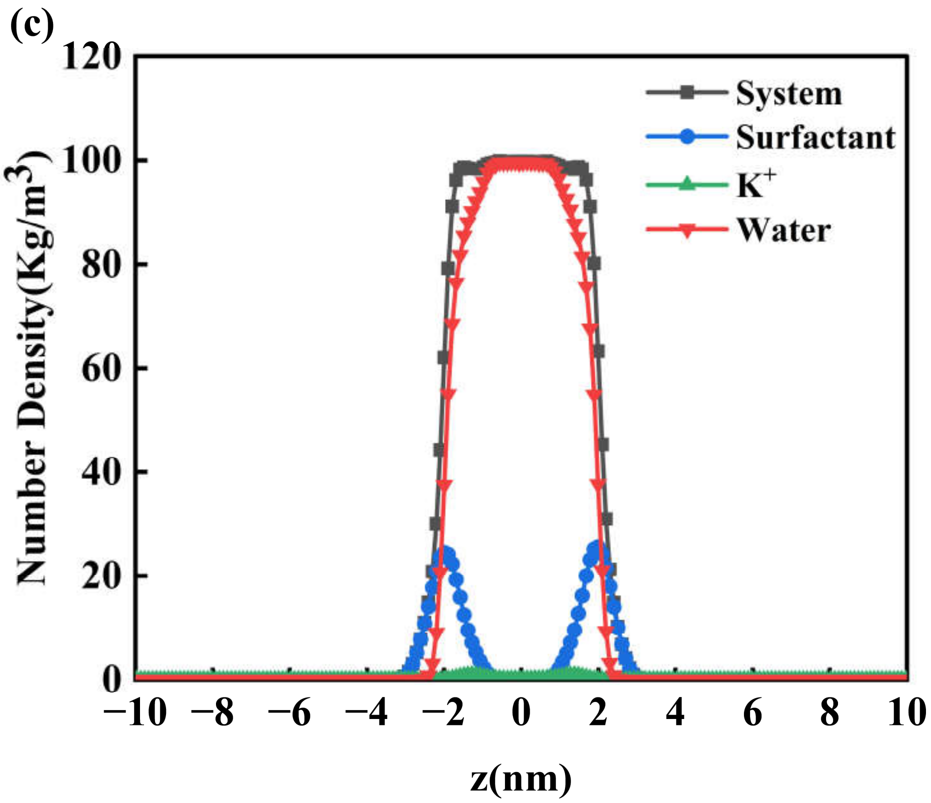

Figure S1. Z-axis Number Density Profiles of PFH$_X$A/SDS Mixed Surfactant Systems and Counterion Distributions: (a-c) PFH$_X$A/SDS Ratios of Li^+, Na^+, K^+.

model of the system

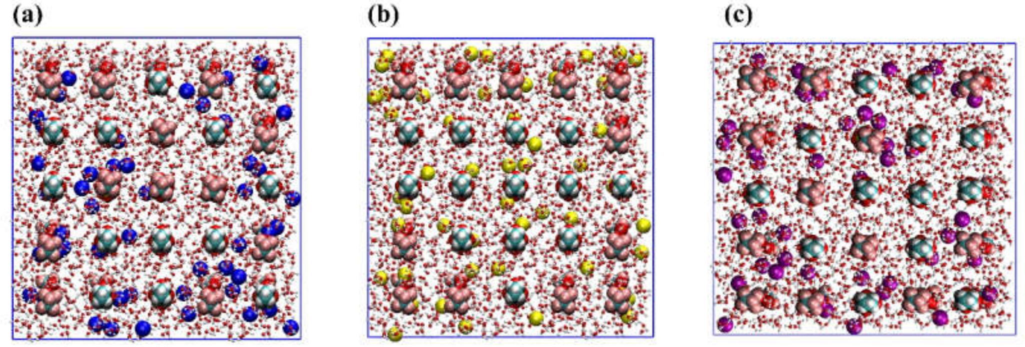

Figure S2. The top views initial model of the system: (a-c) PFH_XA/SDS Ratios of Li^+, Na^+, K^+.

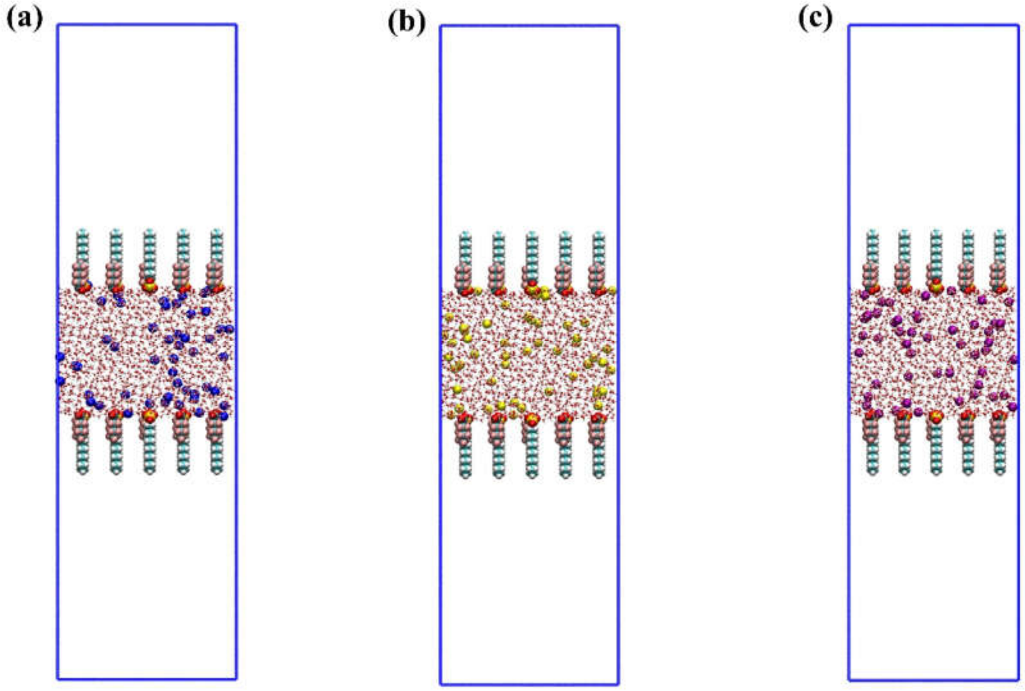

Figure S3. The front views initial model of the system: (a-c) PFH_XA/SDS Ratios of Li^+, Na^+, K^+.

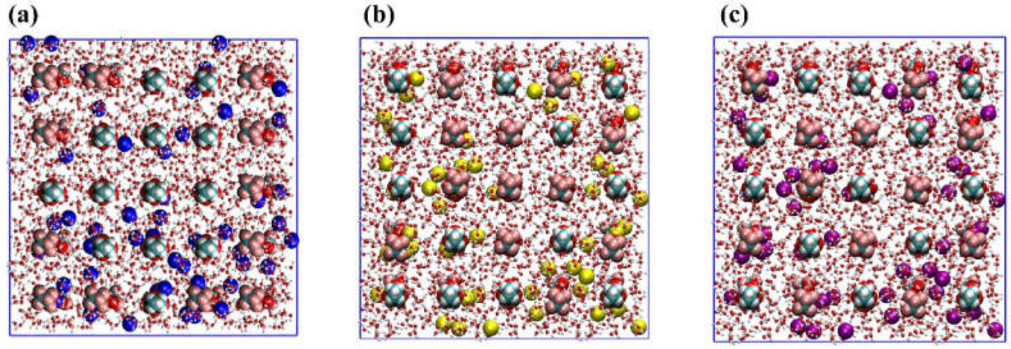

Figure S4. The top view initial model of the recreate system: (a-c) PFH_XA/SDS Ratios of Li^+, Na^+, K^+.

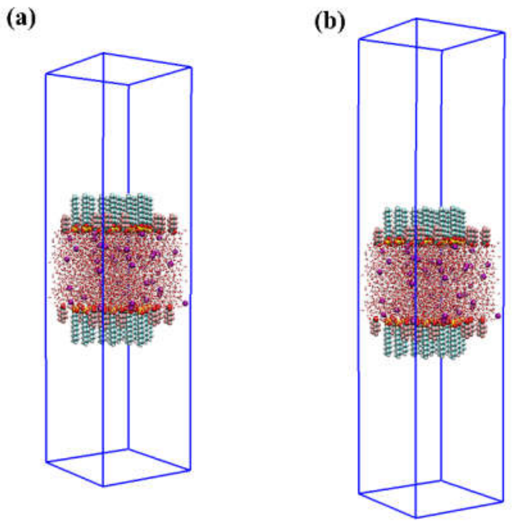

Figure S5. Initial System Model (Z-Axis Dimension of 20 nm) (a) and Enlarged System Model (Z-Axis Dimension of 25 nm) (b): PFH_XA/SDS Ratios of K^+.

RESP Maximum and minimum Distribution of Surfactant

Table S1 RESP Maximum Distribution of Surfactant PFH_XA Ions

maximum	kcal/mol	X(Å)	Y(Å)	Z(Å)
1	-90.441526	-3.457569	-1.666483	1.070191
2	-86.286182	-1.793424	-0.654316	3.157295
3	-72.065471	-1.147691	-0.974294	-1.656539
4	-57.150847	0.027469	0.524927	1.908423
5	-42.249386	1.377731	0.134922	-1.888691
6	-47.102882	2.358627	-2.07857	-0.782204
7	-41.131792	2.959724	2.088707	0.243272
8	-34.21389	3.305456	0.069359	1.768411
9	-35.182362	3.381485	1.343936	-1.341053
10	-27.706962	4.653997	-1.050172	-0.434795
11	-47.495968	5.437433	1.275275	0.357716

Table S2 RESP Minimum Distribution of Surfactant PFBS Ions

minimum	kcal/mol	X(Å)	Y(Å)	Z(Å)
1	-0.21163757	-5.758951	-132.80469	-5.355592
2	-0.2058297	-5.600911	-129.160196	-3.795364
3	-0.20509696	-5.580972	-128.700396	-2.732543
4	-0.08373824	-2.278633	-52.546585	3.805272

Table S3 RESP Maximum Distribution of Surfactant SDS Ions

maximum	kcal/mol	X(Å)	Y(Å)	Z(Å)
1	-0.02070436	-0.563394	-12.992193	-12.259596
2	-0.02294668	-0.624411	-14.399269	-10.564338
3	-0.02295046	-0.624514	-14.401644	-10.554064
4	-0.02685515	-0.730766	-16.851878	-9.20907
5	-0.02683965	-0.730344	-16.842151	-9.195839
6	-0.0295373	-0.803751	-18.534954	-8.120172
7	-0.02952745	-0.803483	-18.528768	-8.058189
8	-0.03268385	-0.889373	-20.509446	-6.725506
9	-0.03268674	-0.889452	-20.511259	-6.669089
10	-0.03630915	-0.988022	-22.784358	-5.613716
11	-0.03630029	-0.987781	-22.778796	-5.607688
12	-0.04070267	-1.107576	-25.541331	-4.303494
13	-0.04069849	-1.107462	-25.538708	-4.31776
14	-0.04568924	-1.243267	-28.670456	-3.124821
15	-0.04568969	-1.24328	-28.670734	-3.121588
16	-0.0519848	-1.414578	-32.620981	-1.879797
17	-0.05198176	-1.414496	-32.619077	-1.819737
18	-0.05928611	-1.613257	-37.202627	-0.768238
19	-0.05929369	-1.613463	-37.207387	-0.742234
20	-0.1821441	-4.956393	-114.297246	5.607892
21	-0.178387	-4.854157	-111.939628	6.662039
22	-0.18783521	-5.111256	-117.868471	8.098931
23	-0.18780016	-5.110302	-117.846476	8.043336
24	-0.15534	-4.22704	-97.4779	8.545812

Table S4 RESP Minimum Distribution of Surfactant SDS Ions

minimum	kcal/mol	X(Å)	Y(Å)	Z(Å)
1	-0.03445	-0.93735	-21.6158	-11.6191
2	-0.03451	-0.93919	-21.6582	-10.796
3	-0.03451	-0.9392	-21.6584	-10.7831
4	-0.20505	-5.5796	-128.669	5.929655
5	-0.20501	-5.5786	-128.646	5.894115
6	-0.19838	-5.39824	-124.486	7.06313
7	-0.1984	-5.39881	-124.5	7.028385
8	-0.19936	-5.42493	-125.102	7.467786
9	-0.19935	-5.42445	-125.091	7.472617
10	-0.20296	-5.5227	-127.357	8.386073
11	-0.20296916	-5.523072	-127.36518	8.460124

Detailed parameters of topology files

SDS.itp

```
[ atomtypes ]
; name      at.num          mass            charge      ptype         sigma (nm)        epsilon
(kJ/mol)
c3                6       12.010736       0.000000       A          3.399670E-01
4.577296E-01
hc                1         1.007941       0.000000       A          2.649533E-01
6.568880E-02
h1                1         1.007941       0.000000       A          2.471353E-01
6.568880E-02
os                8       15.999405       0.000000       A          3.000012E-01
7.112800E-01
s6               16       32.064787       0.000000       A          3.563595E-01
1.046000E+00
o                 8       15.999405       0.000000       A          2.959922E-01
8.786400E-01

[ moleculetype ]
; name                  nrexcl
SDS             3

[ atoms ]
;   Index      type     residue   resname     atom            cgnr         charge
mass
         1         c3             1          MOL        C1               1     -0.25787533
12.010736
         2         c3             1          MOL        C2               2       0.11738414
12.010736
         3         hc             1          MOL        H3               3       0.06022224
1.007941
         4         hc             1          MOL        H4               4       0.06022224
1.007941
         5         hc             1          MOL        H5               5       0.06022224
1.007941
         6         c3             1          MOL        C6               6     -0.00987108
12.010736
         7         hc             1          MOL        H7               7     -0.01427893
1.007941
         8         hc             1          MOL        H8               8     -0.01427893
1.007941
         9         c3             1          MOL        C9               9     -0.03697414
12.010736
```

10	hc	1	MOL	H10	10	0.00244435	1.007941
11	hc	1	MOL	H11	11	0.00244435	1.007941
12	hc	1	MOL	H12	12	-0.00056846	1.007941
13	hc	1	MOL	H13	13	-0.00056846	1.007941
14	c3	1	MOL	C14	14	0.05197036	12.010736
15	c3	1	MOL	C15	15	0.03087003	12.010736
16	hc	1	MOL	H16	16	-0.01475950	1.007941
17	hc	1	MOL	H17	17	-0.01475950	1.007941
18	c3	1	MOL	C18	18	-0.03877654	12.010736
19	hc	1	MOL	H19	19	-0.00968369	1.007941
20	hc	1	MOL	H20	20	-0.00968369	1.007941
21	c3	1	MOL	C21	21	0.00843912	12.010736
22	hc	1	MOL	H22	22	0.00110041	1.007941
23	hc	1	MOL	H23	23	0.00110041	1.007941
24	hc	1	MOL	H24	24	-0.00574299	1.007941
25	hc	1	MOL	H25	25	-0.00574299	1.007941
26	c3	1	MOL	C26	26	0.10696641	12.010736
27	c3	1	MOL	C27	27	-0.05563405	12.010736
28	hc	1	MOL	H28	28	-0.02786062	1.007941
29	hc	1	MOL	H29	29	-0.02786062	1.007941
30	c3	1	MOL	C30	30	-0.09655935	12.010736
31	hc	1	MOL	H31	31	-0.00324552	1.007941

32 hc 1 MOL H32 32 -0.00324552
1.007941
33 c3 1 MOL C33 33 0.38951559
12.010736
34 hc 1 MOL H34 34 0.03401807
1.007941
35 hc 1 MOL H35 35 0.03401807
1.007941
36 h1 1 MOL H36 36 -0.02070795
1.007941
37 h1 1 MOL H37 37 -0.02070795
1.007941
38 os 1 MOL O38 38 -0.58081253
15.999405
39 s6 1 MOL S39 39 1.11657032
32.064787
40 o 1 MOL O40 40 -0.59228433
15.999405
41 o 1 MOL O41 41 -0.59219254
15.999405
42 o 1 MOL O42 42 -0.62283309
15.999405

[bonds]
; atom_i atom_j functype r0 (nm) k (kJ/mol/nm^2)
1 2 1 0.153727 2.670598E+05 ; C1-C2,
DRIH method
1 3 1 0.109658 3.169798E+05 ; C1-H3,
DRIH method
1 4 1 0.109749 3.154878E+05 ; C1-H4,
DRIH method
1 5 1 0.109749 3.154887E+05 ; C1-H5,
DRIH method
2 6 1 0.153985 2.618194E+05 ; C2-C6,
DRIH method
2 7 1 0.110016 3.072732E+05 ; C2-H7,
DRIH method
2 8 1 0.110016 3.072750E+05 ; C2-H8,
DRIH method
6 9 1 0.153943 2.617181E+05 ; C6-C9,
DRIH method
6 10 1 0.110115 3.053242E+05 ; C6-H10,
DRIH method
6 11 1 0.110115 3.053245E+05 ; C6-H11,

DRIH method
9 12 1 0.110108 3.054150E+05 ; C9-H12,
DRIH method
9 13 1 0.110108 3.054147E+05 ; C9-H13,
DRIH method
9 14 1 0.153961 2.616041E+05 ; C9-C14,
DRIH method
14 15 1 0.153959 2.615017E+05 ; C14-
C15, DRIH method
14 16 1 0.110106 3.054221E+05 ; C14-
H16, DRIH method
14 17 1 0.110106 3.054197E+05 ; C14-
H17, DRIH method
15 18 1 0.153961 2.614481E+05 ; C15-
C18, DRIH method
15 19 1 0.110109 3.053567E+05 ; C15-
H19, DRIH method
15 20 1 0.110109 3.053590E+05 ; C15-
H20, DRIH method
18 21 1 0.153965 2.612678E+05 ; C18-
C21, DRIH method
18 22 1 0.110112 3.052626E+05 ; C18-H22,
DRIH method
18 23 1 0.110113 3.052623E+05 ; C18-H23,
DRIH method
21 24 1 0.110115 3.052074E+05 ; C21-H24,
DRIH method
21 25 1 0.110115 3.052063E+05 ; C21-H25,
DRIH method
21 26 1 0.153964 2.611507E+05 ; C21-C26,
DRIH method
26 27 1 0.153960 2.615721E+05 ; C26-
C27, DRIH method
26 28 1 0.110104 3.054115E+05 ; C26-H28,
DRIH method
26 29 1 0.110105 3.054096E+05 ; C26-
H29, DRIH method
27 30 1 0.153982 2.606699E+05 ; C27-
C30, DRIH method
27 31 1 0.110105 3.053333E+05 ; C27-
H31, DRIH method
27 32 1 0.110104 3.053360E+05 ; C27-
H32, DRIH method
30 33 1 0.153078 2.661842E+05 ; C30-

```
C33, DRIH method
    30      34        1       0.109839    3.116999E+05      ; C30-
H34, DRIH method
    30      35        1       0.109839    3.116989E+05      ; C30-
H35, DRIH method
    33      36        1       0.109820    3.081160E+05      ; C33-
H36, DRIH method
    33      37        1       0.109820    3.081193E+05      ; C33-
H37, DRIH method
    33      38        1       0.145086    2.801342E+05      ; C33-
O38, DRIH method
    38      39        1       0.189082    1.148523E+05      ; O38-S39,
DRIH method
    39      40        1       0.163394    2.943010E+05      ; S39-O40,
DRIH method
    39      41        1       0.163394    2.943057E+05      ; S39-O41,
DRIH method
    39      42        1       0.164306    2.826537E+05      ; S39-O42,
DRIH method

[ angles ]
; atom_i   atom_j   atom_k   functype    a0 (Deg.)   k (kJ/mol/rad^2)
     2       1       3       1       111.513      2.776096E+02      ;
C2-C1-H3, DRIH method
     2       1       4       1       111.005      2.827689E+02      ;
C2-C1-H4, DRIH method
     2       1       5       1       111.005      2.827626E+02      ;
C2-C1-H5, DRIH method
     3       1       4       1       107.786      1.433290E+02      ;
H3-C1-H4, DRIH method
     3       1       5       1       107.787      1.433292E+02      ;
H3-C1-H5, DRIH method
     4       1       5       1       107.573      1.430323E+02      ;
H4-C1-H5, DRIH method
     1       2       6       1       113.160      3.537090E+02      ;
C1-C2-C6, DRIH method
     1       2       7       1       109.510      1.321736E+02      ;
C1-C2-H7, DRIH method
     1       2       8       1       109.510      1.321758E+02      ;
C1-C2-H8, DRIH method
     6       2       7       1       109.131      1.331859E+02      ;
C6-C2-H7, DRIH method
     6       2       8       1       109.132      1.331848E+02      ;
C6-C2-H8, DRIH method
```

7 2 8 1 106.157 5.016246E+01 ;
H7-C2-H8, DRIH method
2 6 9 1 113.525 3.514448E+02 ;
C2-C6-C9, DRIH method
2 6 10 1 109.179 1.326393E+02 ;
C2-C6-H10, DRIH method
2 6 11 1 109.179 1.326384E+02 ;
C2-C6-H11, DRIH method
9 6 10 1 109.272 1.327697E+02 ;
C9-C6-H10, DRIH method
9 6 11 1 109.272 1.327684E+02 ;
C9-C6-H11, DRIH method
10 6 11 1 106.149 4.987253E+01 ;
H10-C6-H11, DRIH method
6 9 12 1 109.230 1.328741E+02 ;
C6-C9-H12, DRIH method
6 9 13 1 109.230 1.328761E+02 ;
C6-C9-H13, DRIH method
6 9 14 1 113.526 3.507229E+02 ;
C6-C9-C14, DRIH method
12 9 13 1 106.158 4.986004E+01 ;
H12-C9-H13, DRIH method
12 9 14 1 109.217 1.327033E+02 ;
H12-C9-C14, DRIH method
13 9 14 1 109.217 1.327065E+02 ;
H13-C9-C14, DRIH method
9 14 15 1 113.510 3.517809E+02 ;
C9-C14-C15, DRIH method
9 14 16 1 109.239 1.327651E+02 ;
C9-C14-H16, DRIH method
9 14 17 1 109.240 1.327683E+02 ;
C9-C14-H17, DRIH method
15 14 16 1 109.216 1.330158E+02 ;
C15-C14-H16, DRIH method
15 14 17 1 109.217 1.330170E+02 ;
C15-C14-H17, DRIH method
16 14 17 1 106.157 4.989178E+01 ;
H16-C14-H17, DRIH method
14 15 18 1 113.544 3.514185E+02 ;
C14-C15-C18, DRIH method
14 15 19 1 109.230 1.331187E+02 ;
C14-C15-H19, DRIH method
14 15 20 1 109.230 1.331220E+02 ;
C14-C15-H20, DRIH method

18 15 19 1 109.211 1.326986E+02 ;
C18-C15-H19, DRIH method
18 15 20 1 109.212 1.326998E+02 ;
C18-C15-H20, DRIH method
19 15 20 1 106.148 4.986781E+01 ;
H19-C15-H20, DRIH method
15 18 21 1 113.532 3.518349E+02 ;
C15-C18-C21, DRIH method
15 18 22 1 109.241 1.328517E+02 ;
C15-C18-H22, DRIH method
15 18 23 1 109.240 1.328499E+02 ;
C15-C18-H23, DRIH method
21 18 22 1 109.208 1.328644E+02 ;
C21-C18-H22, DRIH method
21 18 23 1 109.208 1.328652E+02 ;
C21-C18-H23, DRIH method
22 18 23 1 106.147 4.988629E+01 ;
H22-C18-H23, DRIH method
18 21 24 1 109.227 1.331669E+02 ;
C18-C21-H24, DRIH method
18 21 25 1 109.228 1.331696E+02 ;
C18-C21-H25, DRIH method
18 21 26 1 113.576 3.512250E+02 ;
C18-C21-C26, DRIH method
24 21 25 1 106.137 4.984471E+01 ;
H24-C21-H25, DRIH method
24 21 26 1 109.202 1.324392E+02 ;
H24-C21-C26, DRIH method
25 21 26 1 109.202 1.324420E+02 ;
H25-C21-C26, DRIH method
21 26 27 1 113.529 3.524159E+02 ;
C21-C26-C27, DRIH method
21 26 28 1 109.274 1.326522E+02 ;
C21-C26-H28, DRIH method
21 26 29 1 109.274 1.326572E+02 ;
C21-C26-H29, DRIH method
27 26 28 1 109.174 1.327108E+02 ;
C27-C26-H28, DRIH method
27 26 29 1 109.175 1.327111E+02 ;
C27-C26-H29, DRIH method
28 26 29 1 106.151 4.991992E+01 ;
H28-C26-H29, DRIH method
26 27 30 1 113.282 3.486095E+02 ;
C26-C27-C30, DRIH method

26 27 31 1 109.185 1.328595E+02 ;
C26-C27-H31, DRIH method
26 27 32 1 109.185 1.328620E+02 ;
C26-C27-H32, DRIH method
30 27 31 1 109.385 1.310722E+02 ;
C30-C27-H31, DRIH method
30 27 32 1 109.386 1.310731E+02 ;
C30-C27-H32, DRIH method
31 27 32 1 106.170 4.998243E+01 ;
H31-C27-H32, DRIH method
27 30 33 1 112.653 3.446953E+02 ;
C27-C30-C33, DRIH method
27 30 34 1 110.050 1.261131E+02 ;
C27-C30-H34, DRIH method
27 30 35 1 110.050 1.261134E+02 ;
C27-C30-H35, DRIH method
33 30 34 1 108.609 1.303667E+02 ;
C33-C30-H34, DRIH method
33 30 35 1 108.609 1.303686E+02 ;
C33-C30-H35, DRIH method
34 30 35 1 106.675 5.061107E+01 ;
H34-C30-H35, DRIH method
30 33 36 1 110.675 1.881884E+02 ;
C30-C33-H36, DRIH method
30 33 37 1 110.675 1.881844E+02 ;
C30-C33-H37, DRIH method
30 33 38 1 106.385 4.000860E+02 ;
C30-C33-O38, DRIH method
36 33 37 1 107.127 7.936205E+01 ;
H36-C33-H37, DRIH method
36 33 38 1 111.014 1.541018E+02 ;
H36-C33-O38, DRIH method
37 33 38 1 111.015 1.541019E+02 ;
H37-C33-O38, DRIH method
33 38 39 1 113.132 6.683723E+02 ;
C33-O38-S39, DRIH method
38 39 40 1 102.727 4.396403E+02 ;
O38-S39-O40, DRIH method
38 39 41 1 102.725 4.396708E+02 ;
O38-S39-O41, DRIH method
38 39 42 1 103.377 6.270696E+02 ;
O38-S39-O42, DRIH method
40 39 41 1 115.550 2.832937E+02 ;
O40-S39-O41, DRIH method

40 39 42 1 114.928 2.823754E+02 ;
O40-S39-O42, DRIH method
41 39 42 1 114.929 2.823735E+02 ;
O41-S39-O42, DRIH method

[angles] ; bond-bond cross term
; atom_i atom_j atom_k functype r0_ij (nm) r0_jk (nm) k
(kJ/mol/nm^2)
2 1 3 3 0.153727 0.109658
4.077426E+03 ; C2-C1-H3
2 1 4 3 0.153727 0.109749
4.891827E+03 ; C2-C1-H4
2 1 5 3 0.153727 0.109749
4.891841E+03 ; C2-C1-H5
3 1 4 3 0.109658 0.109749
2.097844E+03 ; H3-C1-H4
3 1 5 3 0.109658 0.109749
2.097817E+03 ; H3-C1-H5
4 1 5 3 0.109749 0.109749
2.095536E+03 ; H4-C1-H5
1 2 6 3 0.153727 0.153985
7.876479E+03 ; C1-C2-C6
1 2 7 3 0.153727 0.110016
4.901173E+03 ; C1-C2-H7
1 2 8 3 0.153727 0.110016
4.901093E+03 ; C1-C2-H8
6 2 7 3 0.153985 0.110016
5.159592E+03 ; C6-C2-H7
6 2 8 3 0.153985 0.110016
5.159629E+03 ; C6-C2-H8
7 2 8 3 0.110016 0.110016
2.864910E+03 ; H7-C2-H8
2 6 9 3 0.153985 0.153943
7.916531E+03 ; C2-C6-C9
2 6 10 3 0.153985 0.110115
4.931684E+03 ; C2-C6-H10
2 6 11 3 0.153985 0.110115
4.931614E+03 ; C2-C6-H11
9 6 10 3 0.153943 0.110115
5.002771E+03 ; C9-C6-H10
9 6 11 3 0.153943 0.110115
5.002311E+03 ; C9-C6-H11
10 6 11 3 0.110115 0.110115
2.736213E+03 ; H10-C6-H11

6 9 12 3 0.153943 0.110108
4.975547E+03 ; C6-C9-H12
6 9 13 3 0.153943 0.110108
4.975191E+03 ; C6-C9-H13
6 9 14 3 0.153943 0.153961
7.928884E+03 ; C6-C9-C14
12 9 13 3 0.110108 0.110108
2.738034E+03 ; H12-C9-H13
12 9 14 3 0.110108 0.153961
5.017865E+03 ; H12-C9-C14
13 9 14 3 0.110108 0.153961
5.018057E+03 ; H13-C9-C14
9 14 15 3 0.153961 0.153959
7.940129E+03 ; C9-C14-C15
9 14 16 3 0.153961 0.110106
4.947045E+03 ; C9-C14-H16
9 14 17 3 0.153961 0.110106
4.946876E+03 ; C9-C14-H17
15 14 16 3 0.153959 0.110106
5.046004E+03 ; C15-C14-H16
15 14 17 3 0.153959 0.110106
5.045958E+03 ; C15-C14-H17
16 14 17 3 0.110106 0.110106
2.741991E+03 ; H16-C14-H17
14 15 18 3 0.153959 0.153961
7.923220E+03 ; C14-C15-C18
14 15 19 3 0.153959 0.110109
4.957010E+03 ; C14-C15-H19
14 15 20 3 0.153959 0.110109
4.957252E+03 ; C14-C15-H20
18 15 19 3 0.153961 0.110109
5.036033E+03 ; C18-C15-H19
18 15 20 3 0.153961 0.110109
5.035755E+03 ; C18-C15-H20
19 15 20 3 0.110109 0.110109
2.744184E+03 ; H19-C15-H20
15 18 21 3 0.153961 0.153965
7.940297E+03 ; C15-C18-C21
15 18 22 3 0.153961 0.110112
4.934161E+03 ; C15-C18-H22
15 18 23 3 0.153961 0.110113
4.934136E+03 ; C15-C18-H23
21 18 22 3 0.153965 0.110112
5.070815E+03 ; C21-C18-H22

21 18 23 3 0.153965 0.110113

5.070372E+03 ; C21-C18-H23

22 18 23 3 0.110112 0.110113

2.750272E+03 ; H22-C18-H23

18 21 24 3 0.153965 0.110115

4.935030E+03 ; C18-C21-H24

18 21 25 3 0.153965 0.110115

4.934880E+03 ; C18-C21-H25

18 21 26 3 0.153965 0.153964

7.915298E+03 ; C18-C21-C26

24 21 25 3 0.110115 0.110115

2.752919E+03 ; H24-C21-H25

24 21 26 3 0.110115 0.153964

5.055986E+03 ; H24-C21-C26

25 21 26 3 0.110115 0.153964

5.055869E+03 ; H25-C21-C26

21 26 27 3 0.153964 0.153960

8.008297E+03 ; C21-C26-C27

21 26 28 3 0.153964 0.110104

4.901017E+03 ; C21-C26-H28

21 26 29 3 0.153964 0.110105

4.900995E+03 ; C21-C26-H29

27 26 28 3 0.153960 0.110104

5.069469E+03 ; C27-C26-H28

27 26 29 3 0.153960 0.110105

5.069369E+03 ; C27-C26-H29

28 26 29 3 0.110104 0.110105

2.749555E+03 ; H28-C26-H29

26 27 30 3 0.153960 0.153982

7.832277E+03 ; C26-C27-C30

26 27 31 3 0.153960 0.110105

4.909099E+03 ; C26-C27-H31

26 27 32 3 0.153960 0.110104

4.909512E+03 ; C26-C27-H32

30 27 31 3 0.153982 0.110105

5.310456E+03 ; C30-C27-H31

30 27 32 3 0.153982 0.110104

5.309839E+03 ; C30-C27-H32

31 27 32 3 0.110105 0.110104

2.831492E+03 ; H31-C27-H32

27 30 33 3 0.153982 0.153078

8.233886E+03 ; C27-C30-C33

27 30 34 3 0.153982 0.109839

4.371672E+03 ; C27-C30-H34

27 30 35 3 0.153982 0.109839
4.371580E+03 ; C27-C30-H35
33 30 34 3 0.153078 0.109839
4.480751E+03 ; C33-C30-H34
33 30 35 3 0.153078 0.109839
4.480926E+03 ; C33-C30-H35
34 30 35 3 0.109839 0.109839
2.513451E+03 ; H34-C30-H35
30 33 36 3 0.153078 0.109820
4.186274E+03 ; C30-C33-H36
30 33 37 3 0.153078 0.109820
4.186152E+03 ; C30-C33-H37
30 33 38 3 0.153078 0.145086
1.649022E+04 ; C30-C33-O38
36 33 37 3 0.109820 0.109820
3.682514E+03 ; H36-C33-H37
36 33 38 3 0.109820 0.145086
1.319827E+04 ; H36-C33-O38
37 33 38 3 0.109820 0.145086
1.319722E+04 ; H37-C33-O38
33 38 39 3 0.145086 0.189082
1.760604E+04 ; C33-O38-S39
38 39 40 3 0.189082 0.163394
3.953157E+03 ; O38-S39-O40
38 39 41 3 0.189082 0.163394
3.952643E+03 ; O38-S39-O41
38 39 42 3 0.189082 0.164306
7.585390E+03 ; O38-S39-O42
40 39 41 3 0.163394 0.163394
8.644500E+03 ; O40-S39-O41
40 39 42 3 0.163394 0.164306
8.003586E+03 ; O40-S39-O42
41 39 42 3 0.163394 0.164306
8.002934E+03 ; O41-S39-O42

[dihedrals] ; propers
; atom_i atom_j atom_k atom_l functype d0 (Deg.) k
(kJ/mol/rad^2) ; Case of functype=2
; atom_i atom_j atom_k atom_l functype phase (Deg.) kd (kJ/mol)
pn ; Case of functype=9
1 2 6 9 2 -179.999
3.730266E+01 ; C1-C2-C6-C9, DRIH method
1 2 6 10 2 -57.821
3.879407E+01 ; C1-C2-C6-H10, DRIH method

1 2 6 11 2 57.822
3.879532E+01 ; C1-C2-C6-H11, DRIH method
2 6 9 12 2 -57.854
3.864634E+01 ; C2-C6-C9-H12, DRIH method
2 6 9 13 2 57.856
3.864554E+01 ; C2-C6-C9-H13, DRIH method
2 6 9 14 2 -179.998
3.741211E+01 ; C2-C6-C9-C14, DRIH method
3 1 2 6 2 -179.991
3.869429E+01 ; H3-C1-C2-C6, DRIH method
3 1 2 7 2 -58.003
4.051704E+01 ; H3-C1-C2-H7, DRIH method
3 1 2 8 2 58.021
4.051645E+01 ; H3-C1-C2-H8, DRIH method
4 1 2 6 2 -59.788
3.840104E+01 ; H4-C1-C2-C6, DRIH method
4 1 2 7 2 62.199
4.227779E+01 ; H4-C1-C2-H7, DRIH method
4 1 2 8 2 178.224
3.997019E+01 ; H4-C1-C2-H8, DRIH method
5 1 2 6 2 59.806
3.840082E+01 ; H5-C1-C2-C6, DRIH method
5 1 2 7 2 -178.206
3.997138E+01 ; H5-C1-C2-H7, DRIH method
5 1 2 8 2 -62.182
4.227683E+01 ; H5-C1-C2-H8, DRIH method
6 9 14 15 2 179.993
3.813157E+01 ; C6-C9-C14-C15, DRIH method
6 9 14 16 2 57.854
3.889943E+01 ; C6-C9-C14-H16, DRIH method
6 9 14 17 2 -57.866
3.889802E+01 ; C6-C9-C14-H17, DRIH method
7 2 6 9 2 57.801
3.911590E+01 ; H7-C2-C6-C9, DRIH method
7 2 6 10 2 179.980
3.995633E+01 ; H7-C2-C6-H10, DRIH method
7 2 6 11 2 -64.377
4.260776E+01 ; H7-C2-C6-H11, DRIH method
8 2 6 9 2 -57.799
3.911507E+01 ; H8-C2-C6-C9, DRIH method
8 2 6 10 2 64.379
4.260725E+01 ; H8-C2-C6-H10, DRIH method
8 2 6 11 2 -179.978
3.995612E+01 ; H8-C2-C6-H11, DRIH method

9 14 15 18 2 -179.997
3.825049E+01 ; C9-C14-C15-C18, DRIH method
9 14 15 19 2 -57.847
3.892171E+01 ; C9-C14-C15-H19, DRIH method
9 14 15 20 2 57.852
3.892045E+01 ; C9-C14-C15-H20, DRIH method
10 6 9 12 2 -179.981
3.991071E+01 ; H10-C6-C9-H12, DRIH method
10 6 9 13 2 -64.271
4.249935E+01 ; H10-C6-C9-H13, DRIH method
10 6 9 14 2 57.875
3.892287E+01 ; H10-C6-C9-C14, DRIH method
11 6 9 12 2 64.272
4.250002E+01 ; H11-C6-C9-H12, DRIH method
11 6 9 13 2 179.982
3.991013E+01 ; H11-C6-C9-H13, DRIH method
11 6 9 14 2 -57.872
3.892347E+01 ; H11-C6-C9-C14, DRIH method
12 9 14 15 2 57.842
3.913670E+01 ; H12-C9-C14-C15, DRIH method
12 9 14 16 2 -64.298
4.251771E+01 ; H12-C9-C14-H16, DRIH method
12 9 14 17 2 179.983
3.993128E+01 ; H12-C9-C14-H17, DRIH method
13 9 14 15 2 -57.854
3.913657E+01 ; H13-C9-C14-C15, DRIH method
13 9 14 16 2 -179.993
3.993101E+01 ; H13-C9-C14-H16, DRIH method
13 9 14 17 2 64.287
4.251635E+01 ; H13-C9-C14-H17, DRIH method
14 15 18 21 2 179.997
3.848951E+01 ; C14-C15-C18-C21, DRIH method
14 15 18 22 2 -57.858
3.903057E+01 ; C14-C15-C18-H22, DRIH method
14 15 18 23 2 57.852
3.903178E+01 ; C14-C15-C18-H23, DRIH method
15 18 21 24 2 -57.843
3.894059E+01 ; C15-C18-C21-H24, DRIH method
15 18 21 25 2 57.840
3.893989E+01 ; C15-C18-C21-H25, DRIH method
15 18 21 26 2 179.999
3.814582E+01 ; C15-C18-C21-C26, DRIH method
16 14 15 18 2 -57.845
3.902115E+01 ; H16-C14-C15-C18, DRIH method

16 14 15 19 2 64.306
4.251219E+01 ; H16-C14-C15-H19, DRIH method
16 14 15 20 2 -179.996
3.994149E+01 ; H16-C14-C15-H20, DRIH method
17 14 15 18 2 57.849
3.902065E+01 ; H17-C14-C15-C18, DRIH method
17 14 15 19 2 180.000
3.994237E+01 ; H17-C14-C15-H19, DRIH method
17 14 15 20 2 -64.302
4.251115E+01 ; H17-C14-C15-H20, DRIH method
18 21 26 27 2 179.991
3.820156E+01 ; C18-C21-C26-C27, DRIH method
18 21 26 28 2 57.868
3.901677E+01 ; C18-C21-C26-H28, DRIH method
18 21 26 29 2 -57.883
3.901602E+01 ; C18-C21-C26-H29, DRIH method
19 15 18 21 2 57.836
3.909329E+01 ; H19-C15-C18-C21, DRIH method
19 15 18 22 2 179.982
3.990718E+01 ; H19-C15-C18-H22, DRIH method
19 15 18 23 2 -64.309
4.249812E+01 ; H19-C15-C18-H23, DRIH method
20 15 18 21 2 -57.842
3.909277E+01 ; H20-C15-C18-C21, DRIH method
20 15 18 22 2 64.304
4.249701E+01 ; H20-C15-C18-H22, DRIH method
20 15 18 23 2 -179.987
3.990698E+01 ; H20-C15-C18-H23, DRIH method
21 26 27 30 2 -179.999
3.736855E+01 ; C21-C26-C27-C30, DRIH method
21 26 27 31 2 -57.838
3.881718E+01 ; C21-C26-C27-H31, DRIH method
21 26 27 32 2 57.838
3.881615E+01 ; C21-C26-C27-H32, DRIH method
22 18 21 24 2 179.993
3.988879E+01 ; H22-C18-C21-H24, DRIH method
22 18 21 25 2 -64.323
4.246887E+01 ; H22-C18-C21-H25, DRIH method
22 18 21 26 2 57.835
3.885352E+01 ; H22-C18-C21-C26, DRIH method
23 18 21 24 2 64.319
4.246923E+01 ; H23-C18-C21-H24, DRIH method
23 18 21 25 2 -179.997
3.988840E+01 ; H23-C18-C21-H25, DRIH method

23 18 21 26 2 -57.838
3.885386E+01 ; H23-C18-C21-C26, DRIH method
24 21 26 27 2 57.820
3.892693E+01 ; H24-C21-C26-C27, DRIH method
24 21 26 28 2 -64.304
4.245160E+01 ; H24-C21-C26-H28, DRIH method
24 21 26 29 2 179.945
3.983998E+01 ; H24-C21-C26-H29, DRIH method
25 21 26 27 2 -57.835
3.892611E+01 ; H25-C21-C26-C27, DRIH method
25 21 26 28 2 -179.959
3.984009E+01 ; H25-C21-C26-H28, DRIH method
25 21 26 29 2 64.290
4.245002E+01 ; H25-C21-C26-H29, DRIH method
26 27 30 33 2 179.996
3.753037E+01 ; C26-C27-C30-C33, DRIH method
26 27 30 34 2 -58.644
3.896384E+01 ; C26-C27-C30-H34, DRIH method
26 27 30 35 2 58.637
3.896531E+01 ; C26-C27-C30-H35, DRIH method
27 30 33 36 2 -59.306
4.123483E+01 ; C27-C30-C33-H36, DRIH method
27 30 33 37 2 59.303
4.123355E+01 ; C27-C30-C33-H37, DRIH method
27 30 33 38 2 179.999
4.461772E+01 ; C27-C30-C33-O38, DRIH method
28 26 27 30 2 -57.820
3.861222E+01 ; H28-C26-C27-C30, DRIH method
28 26 27 31 2 64.341
4.243918E+01 ; H28-C26-C27-H31, DRIH method
28 26 27 32 2 -179.983
3.986041E+01 ; H28-C26-C27-H32, DRIH method
29 26 27 30 2 57.821
3.861180E+01 ; H29-C26-C27-C30, DRIH method
29 26 27 31 2 179.982
3.986066E+01 ; H29-C26-C27-H31, DRIH method
29 26 27 32 2 -64.342
4.243852E+01 ; H29-C26-C27-H32, DRIH method
30 33 38 39 2 -179.988
4.899157E+01 ; C30-C33-O38-S39, DRIH method
31 27 30 33 2 57.946
3.899089E+01 ; H31-C27-C30-C33, DRIH method
31 27 30 34 2 179.306
3.998878E+01 ; H31-C27-C30-H34, DRIH method

31 27 30 35 2 -63.413
4.262268E+01 ; H31-C27-C30-H35, DRIH method
32 27 30 33 2 -57.953
3.899005E+01 ; H32-C27-C30-C33, DRIH method
32 27 30 34 2 63.406
4.262141E+01 ; H32-C27-C30-H34, DRIH method
32 27 30 35 2 -179.312
3.998920E+01 ; H32-C27-C30-H35, DRIH method
33 38 39 40 2 119.840
8.970097E+01 ; C33-O38-S39-O40, DRIH method
33 38 39 41 2 -119.876
8.970976E+01 ; C33-O38-S39-O41, DRIH method
33 38 39 42 2 -0.018
8.792772E+01 ; C33-O38-S39-O42, DRIH method
34 30 33 36 2 178.518
4.202139E+01 ; H34-C30-C33-H36, DRIH method
34 30 33 37 2 -62.873
4.584188E+01 ; H34-C30-C33-H37, DRIH method
34 30 33 38 2 57.823
4.538001E+01 ; H34-C30-C33-O38, DRIH method
35 30 33 36 2 62.869
4.584207E+01 ; H35-C30-C33-H36, DRIH method
35 30 33 37 2 -178.521
4.202158E+01 ; H35-C30-C33-H37, DRIH method
35 30 33 38 2 -57.826
4.537951E+01 ; H35-C30-C33-O38, DRIH method
36 33 38 39 2 59.535
6.439067E+01 ; H36-C33-O38-S39, DRIH method
37 33 38 39 2 -59.511
6.438522E+01 ; H37-C33-O38-S39, DRIH method

PFH$_X$A.itp

[atomtypes]

; name	at.num	mass	charge	ptype	sigma (nm)	epsilon (kJ/mol)
c3	6	12.010736	0.000000	A	3.399670E-01	4.577296E-01
f	9	18.998403	0.000000	A	3.118146E-01	2.552240E-01
c	6	12.010736	0.000000	A	3.399670E-01	3.598240E-01
o	8	15.999405	0.000000	A	2.959922E-01	8.786400E-01

[moleculetype]

; name	nrexcl
PFH$_X$A	3

[atoms]

; Index	type	residue	resname	atom	cgnr	charge	mass
1	c3	1	MOL	C1	1	0.65938193	12.010736
2	c3	1	MOL	C2	2	0.20868072	12.010736
3	c3	1	MOL	C3	3	0.41499987	12.010736
4	c3	1	MOL	C4	4	0.28942482	12.010736
5	c3	1	MOL	C5	5	0.33657049	12.010736
6	f	1	MOL	F6	6	-0.21045228	18.998403
7	f	1	MOL	F7	7	-0.21000762	18.998403
8	f	1	MOL	F8	8	-0.20966861	18.998403
9	f	1	MOL	F9	9	-0.15570669	18.998403
10	f	1	MOL	F10	10	-0.16848622	18.998403
11	f	1	MOL	F11	11	-0.20921247	18.998403
12	f	1	MOL	F12	12	-0.19698297	18.998403

```
     13      f       1     MOL     F13        13   -0.18354705
18.998403
     14      f       1     MOL     F14        14   -0.20477902
18.998403
     15      f       1     MOL     F15        15   -0.25375507
18.998403
     16      f       1     MOL     F16        16   -0.24683263
18.998403
     17      c       1     MOL     C17        17    0.74448359
12.010736
     18      o       1     MOL     O18        18   -0.70875001
15.999405
     19      o       1     MOL     O19        19   -0.69536079
15.999405

[ bonds ]
; atom_i   atom_j   functype       r0 (nm)      k (kJ/mol/nm^2)
     1       2        1        0.154385      2.587223E+05      ; C1-C2,
DRIH method
     1       6        1        0.138412      3.449576E+05      ; C1-F6,
DRIH method
     1       7        1        0.137705      3.580219E+05      ; C1-F7,
DRIH method
     1       8        1        0.137982      3.548800E+05      ; C1-F8,
DRIH method
     2       3        1        0.155018      2.519975E+05      ; C2-C3,
DRIH method
     2       9        1        0.139465      3.440676E+05      ; C2-F9,
DRIH method
     2      10        1        0.139306      3.461769E+05      ; C2-F10,
DRIH method
     3       4        1        0.155363      2.431614E+05      ; C3-C4,
DRIH method
     3      11        1        0.139934      3.356723E+05      ; C3-F11,
DRIH method
     3      12        1        0.139629      3.391629E+05      ; C3-F12,
DRIH method
     4       5        1        0.154021      2.491763E+05      ; C4-C5,
DRIH method
     4      13        1        0.139858      3.346371E+05      ; C4-F13,
DRIH method
     4      14        1        0.140090      3.300306E+05      ; C4-F14,
DRIH method
     5      15        1        0.141647      2.976212E+05      ; C5-F15,
```

DRIH method
5 16 1 0.141721 3.021871E+05 ; C5-F16, DRIH method
5 17 1 0.158354 1.617261E+05 ; C5-C17, DRIH method
17 18 1 0.127046 5.910028E+05 ; C17-O18, DRIH method
17 19 1 0.126214 6.124872E+05 ; C17-O19, DRIH method

[angles]
; atom_i atom_j atom_k functype a0 (Deg.) k (kJ/mol/rad^2)
2 1 6 1 109.530 4.842664E+02 ; C2-C1-F6, DRIH method
2 1 7 1 111.777 4.653960E+02 ; C2-C1-F7, DRIH method
2 1 8 1 111.292 4.644317E+02 ; C2-C1-F8, DRIH method
6 1 7 1 107.552 3.017802E+02 ; F6-C1-F7, DRIH method
6 1 8 1 107.825 2.926047E+02 ; F6-C1-F8, DRIH method
7 1 8 1 108.714 2.995705E+02 ; F7-C1-F8, DRIH method
1 2 3 1 115.307 5.296114E+02 ; C1-C2-C3, DRIH method
1 2 9 1 106.166 2.245655E+02 ; C1-C2-F9, DRIH method
1 2 10 1 106.468 2.237951E+02 ; C1-C2-F10, DRIH method
3 2 9 1 110.377 2.017650E+02 ; C3-C2-F9, DRIH method
3 2 10 1 109.536 2.094423E+02 ; C3-C2-F10, DRIH method
9 2 10 1 108.733 8.272421E+01 ; F9-C2-F10, DRIH method
2 3 4 1 115.440 5.287963E+02 ; C2-C3-C4, DRIH method
2 3 11 1 106.506 2.367255E+02 ; C2-C3-F11, DRIH method
2 3 12 1 106.093 2.342774E+02 ; C2-C3-F12, DRIH method
4 3 11 1 109.971 2.120558E+02 ; C4-C3-F11, DRIH method

4 3 12 1 110.332 2.038879E+02 ;
C4-C3-F12, DRIH method
11 3 12 1 108.180 8.336373E+01 ;
F11-C3-F12, DRIH method
3 4 5 1 116.331 5.181101E+02 ;
C3-C4-C5, DRIH method
3 4 13 1 105.593 2.419758E+02 ;
C3-C4-F13, DRIH method
3 4 14 1 105.489 2.308227E+02 ;
C3-C4-F14, DRIH method
5 4 13 1 110.989 2.095912E+02 ;
C5-C4-F13, DRIH method
5 4 14 1 109.425 1.969828E+02 ;
C5-C4-F14, DRIH method
13 4 14 1 108.643 7.963867E+01 ;
F13-C4-F14, DRIH method
4 5 15 1 105.456 2.681969E+02 ;
C4-C5-F15, DRIH method
4 5 16 1 106.319 2.329393E+02 ;
C4-C5-F16, DRIH method
4 5 17 1 115.033 3.828535E+02 ;
C4-C5-C17, DRIH method
15 5 16 1 105.997 9.303725E+01 ;
F15-C5-F16, DRIH method
15 5 17 1 113.083 2.031242E+02 ;
F15-C5-C17, DRIH method
16 5 17 1 110.322 1.567112E+02 ;
F16-C5-C17, DRIH method
5 17 18 1 111.647 4.004102E+02 ;
C5-C17-O18, DRIH method
5 17 19 1 115.106 3.981908E+02 ;
C5-C17-O19, DRIH method
18 17 19 1 133.224 4.887620E+02 ;
O18-C17-O19, DRIH method

[angles] ; bond-bond cross term
; atom_i atom_j atom_k functype r0_ij (nm) r0_jk (nm) k
(kJ/mol/nm^2)
2 1 6 3 0.154385 0.138412
1.382165E+04 ; C2-C1-F6
2 1 7 3 0.154385 0.137705
1.160314E+04 ; C2-C1-F7
2 1 8 3 0.154385 0.137982
1.216627E+04 ; C2-C1-F8

6 1 7 3 0.138412 0.137705
3.882471E+04 ; F6-C1-F7
6 1 8 3 0.138412 0.137982
3.798914E+04 ; F6-C1-F8
7 1 8 3 0.137705 0.137982
3.738043E+04 ; F7-C1-F8
1 2 3 3 0.154385 0.155018
1.821919E+04 ; C1-C2-C3
1 2 9 3 0.154385 0.139465
1.832715E+04 ; C1-C2-F9
1 2 10 3 0.154385 0.139306
1.764728E+04 ; C1-C2-F10
3 2 9 3 0.155018 0.139465
1.266076E+04 ; C3-C2-F9
3 2 10 3 0.155018 0.139306
1.349503E+04 ; C3-C2-F10
9 2 10 3 0.139465 0.139306
3.533633E+04 ; F9-C2-F10
2 3 4 3 0.155018 0.155363
1.802954E+04 ; C2-C3-C4
2 3 11 3 0.155018 0.139934
1.801985E+04 ; C2-C3-F11
2 3 12 3 0.155018 0.139629
1.957235E+04 ; C2-C3-F12
4 3 11 3 0.155363 0.139934
1.149352E+04 ; C4-C3-F11
4 3 12 3 0.155363 0.139629
1.179583E+04 ; C4-C3-F12
11 3 12 3 0.139934 0.139629
3.524968E+04 ; F11-C3-F12
3 4 5 3 0.155363 0.154021
1.864799E+04 ; C3-C4-C5
3 4 13 3 0.155363 0.139858
2.036897E+04 ; C3-C4-F13
3 4 14 3 0.155363 0.140090
2.079703E+04 ; C3-C4-F14
5 4 13 3 0.154021 0.139858
9.905021E+03 ; C5-C4-F13
5 4 14 3 0.154021 0.140090
1.493040E+04 ; C5-C4-F14
13 4 14 3 0.139858 0.140090
3.419778E+04 ; F13-C4-F14
4 5 15 3 0.154021 0.141647
1.759900E+04 ; C4-C5-F15

4 5 16 3 0.154021 0.141721
1.798846E+04 ; C4-C5-F16
4 5 17 3 0.154021 0.158354
1.279757E+04 ; C4-C5-C17
15 5 16 3 0.141647 0.141721
3.357959E+04 ; F15-C5-F16
15 5 17 3 0.141647 0.158354
5.468785E+03 ; F15-C5-C17
16 5 17 3 0.141721 0.158354
7.253260E+03 ; F16-C5-C17
5 17 18 3 0.158354 0.127046
4.705772E+04 ; C5-C17-O18
5 17 19 3 0.158354 0.126214
4.249290E+04 ; C5-C17-O19
18 17 19 3 0.127046 0.126214
5.569632E+04 ; O18-C17-O19

[dihedrals] ; propers
; atom_i atom_j atom_k atom_l functype d0 (Deg.) k
(kJ/mol/rad^2) ; Case of functype=2
; atom_i atom_j atom_k atom_l functype phase (Deg.) kd (kJ/mol)
pn ; Case of functype=9
1 2 3 4 2 -162.176
5.412045E+01 ; C1-C2-C3-C4, DRIH method
1 2 3 11 2 -39.802
6.642954E+01 ; C1-C2-C3-F11, DRIH method
1 2 3 12 2 75.297
6.652788E+01 ; C1-C2-C3-F12, DRIH method
2 3 4 5 2 -162.661
5.490107E+01 ; C2-C3-C4-C5, DRIH method
2 3 4 13 2 -39.085
6.746976E+01 ; C2-C3-C4-F13, DRIH method
2 3 4 14 2 75.859
6.871547E+01 ; C2-C3-C4-F14, DRIH method
3 2 1 6 2 -168.763
7.596564E+01 ; C3-C2-C1-F6, DRIH method
3 2 1 7 2 72.141
7.535797E+01 ; C3-C2-C1-F7, DRIH method
3 2 1 8 2 -49.630
7.021865E+01 ; C3-C2-C1-F8, DRIH method
3 4 5 15 2 -37.650
6.475966E+01 ; C3-C4-C5-F15, DRIH method
3 4 5 16 2 74.614
6.441029E+01 ; C3-C4-C5-F16, DRIH method

3 4 5 17 2 -162.966
5.165929E+01 ; C3-C4-C5-C17, DRIH method
4 3 2 9 2 -41.900
6.830518E+01 ; C4-C3-C2-F9, DRIH method
4 3 2 10 2 77.791
7.055201E+01 ; C4-C3-C2-F10, DRIH method
4 5 17 18 2 -49.068
7.404582E+01 ; C4-C5-C17-O18, DRIH method
4 5 17 19 2 132.437
6.922454E+01 ; C4-C5-C17-O19, DRIH method
5 4 3 11 2 76.833
7.099483E+01 ; C5-C4-C3-F11, DRIH method
5 4 3 12 2 -42.421
6.776174E+01 ; C5-C4-C3-F12, DRIH method
6 1 2 9 2 68.683
8.061965E+01 ; F6-C1-C2-F9, DRIH method
6 1 2 10 2 -47.062
8.487263E+01 ; F6-C1-C2-F10, DRIH method
7 1 2 9 2 -50.413
8.050997E+01 ; F7-C1-C2-F9, DRIH method
7 1 2 10 2 -166.158
8.807710E+01 ; F7-C1-C2-F10, DRIH method
8 1 2 9 2 -172.183
8.335207E+01 ; F8-C1-C2-F9, DRIH method
8 1 2 10 2 72.071
8.140336E+01 ; F8-C1-C2-F10, DRIH method
9 2 3 11 2 80.474
7.705697E+01 ; F9-C2-C3-F11, DRIH method
9 2 3 12 2 -164.427
8.275251E+01 ; F9-C2-C3-F12, DRIH method
10 2 3 11 2 -159.835
8.365192E+01 ; F10-C2-C3-F11, DRIH method
10 2 3 12 2 -44.736
7.377701E+01 ; F10-C2-C3-F12, DRIH method
11 3 4 13 2 -159.591
8.419614E+01 ; F11-C3-C4-F13, DRIH method
11 3 4 14 2 -44.647
7.416646E+01 ; F11-C3-C4-F14, DRIH method
12 3 4 13 2 81.155
7.721035E+01 ; F12-C3-C4-F13, DRIH method
12 3 4 14 2 -163.901
8.362993E+01 ; F12-C3-C4-F14, DRIH method
13 4 5 15 2 -158.388
7.974847E+01 ; F13-C4-C5-F15, DRIH method

13 4 5 16 2 -46.124
6.958147E+01 ; F13-C4-C5-F16, DRIH method
13 4 5 17 2 76.296
6.689778E+01 ; F13-C4-C5-C17, DRIH method
14 4 5 15 2 81.722
7.659092E+01 ; F14-C4-C5-F15, DRIH method
14 4 5 16 2 -166.014
8.150943E+01 ; F14-C4-C5-F16, DRIH method
14 4 5 17 2 -43.594
6.496221E+01 ; F14-C4-C5-C17, DRIH method
15 5 17 18 2 -170.317
9.120235E+01 ; F15-C5-C17-O18, DRIH method
15 5 17 19 2 11.188
9.118248E+01 ; F15-C5-C17-O19, DRIH method
16 5 17 18 2 71.174
9.497806E+01 ; F16-C5-C17-O18, DRIH method
16 5 17 19 2 -107.321
8.154691E+01 ; F16-C5-C17-O19, DRIH method
